# An optimized method for obtaining clinical‐grade specific cell subpopulations from human umbilical cord‐derived mesenchymal stem cells

**DOI:** 10.1111/cpr.13300

**Published:** 2022-06-29

**Authors:** Yali Jia, Ailin Wang, Bichun Zhao, Chao Wang, Ruyu Su, Biao Zhang, Zeng Fan, Quan Zeng, Lijuan He, Xuetao Pei, Wen Yue

**Affiliations:** ^1^ Stem Cell and Regenerative Medicine Lab Beijing Institute of Radiation Medicine Beijing China; ^2^ South China Institute of Biomedicine Guangzhou China; ^3^ Institute of Health Service and Transfusion Medicine Beijing China

## Abstract

Mesenchymal stem cells (MSCs) are heterogeneous populations with broad application prospects in cell therapy, and using specific subpopulations of MSCs can enhance their particular capability under certain conditions and achieve better therapeutic effects. However, no studies have reported how to obtain high‐quality specific MSC subpopulations in vitro culture. Here, for the first time, we established a general operation process for obtaining high‐quality clinical‐grade cell subpopulations from human umbilical cord MSCs (hUC‐MSCs) based on particular markers. We used the MSC‐CD106^+^ subpopulations, whose biological function has been well documented, as an example to explore and optimize the crucial links of primary preparation, pre‐treatment, antibody incubation, flow sorting, quality and function test. After comprehensively evaluating the quality and function of the acquired MSC‐CD106^+^ subpopulations, including in vitro cell viability, apoptosis, proliferation, marker stability, adhesion ability, migration ability, tubule formation ability, immunomodulatory function and in vivo wound healing ability and proangiogenic activity, we defined an important pre‐treatment scheme which might effectively improve the therapeutic efficiency of MSC‐CD106^+^ subpopulations in two critical clinical application scenarios—direct injection after cell sorting and post‐culture injection into bodies. Based on the above, we tried to establish a general five‐step operation procedure for acquiring high‐quality clinical‐grade MSC subpopulations based on specific markers, which cannot only improve their enrichment efficiency and the reliability of preclinical studies, but also provide valuable methodological guidance for the rapid clinical transformation of specific MSC subpopulations.

## Graphical abstract

We established an optimized operation process for obtaining human umbilical cord mesenchymal stem cells (hUC‐MSC) subpopulations with specific markers, which is also suitable for MSCs derived from other tissues.

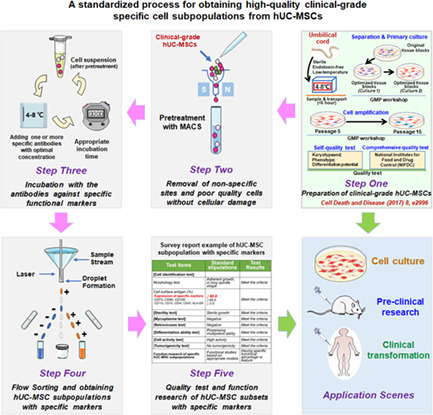

*Step One*. Preparation of clinical‐grade hUC‐MSCs. The clinical‐grade hUC‐MSCs obtained fully comply with the cGMP guidelines and meet the quality standards of the NIFDC. *Step Two*. Removal of non‐specific sites and poor‐quality cells without cellular damage. Treatment with MACS was proved as a safe and effective pre‐treatment by verifying multiple core indicators related to subpopulation quality. *Step Three*. Incubation with the antibodies against specific functional markers. Select the optimum condition of specific antibodies to ensure the purity of subsets obtained by subsequent sorting. *Step Four*. Flow Sorting and obtaining hUC‐MSC subpopulations with specific markers. This step has higher technical requirements related to flow cytometry. *Step Five*. Quality test and function research of hUC‐MSC subpopulations with specific markers. Do more research and verification to ensure the efficiency of clinical transformation of the obtained MSC subpopulations. *Application Scenes*. The clinical‐grade MSC subpopulations obtained through the above‐optimized operation process do not only guarantee the quality of the cells in basic research and preclinical research but also provide safety assurance for clinical application.

## 1 INTRODUCTION

Mesenchymal stem cells (MSCs) have been considered an ideal source for stem cell therapy strategies. MSCs can be obtained from multiple tissues such as bone marrow, adipose tissue, dental pulp, umbilical cord and even human pluripotent stem cells.^1^, ^2^, ^3^ For decades, our group and many other investigators have certified many benefits of MSC‐based interventions for a range of degenerative and inflammatory diseases including, neurological disorders, diabetes and osteochondral defects.^4^, ^5^, ^6^, ^7^, ^8^, ^9^ Compared to the adult tissue‐derived counterparts, human umbilical cord‐derived MSCs (hUC‐MSCs) have a higher yield without invasive procedures and ethical issues; notably, they maintain an earlier embryologic phase, are much younger and can secrete a wide range of multifunctional factors. These characteristics indicate that hUC‐MSCs may be a better choice for clinical application than many other MSCs.^4^, ^10^, ^11^

As with MSCs from all sources, hUC‐MSCs are heterogenous populations consisting of cells with distinct morphology, functions and features,^12^, ^13^, ^14^ which may also impair their therapeutic efficacy and introduce variations between studies.^13^ The use of specific subpopulations of MSCs may eliminate some interfering cells to enhance their particular capability under certain conditions and achieve more effective therapies.^12^, ^15^, ^16^, ^17^, ^18^ Therefore, it is crucial to characterize the differences of hUC‐MSC sub‐populations concerning differentiation and proliferation potential, immunosuppressive ability and other latent biological functions to compare studies and standardized therapies. Although obtaining a subpopulation of MSCs with specific functional advantages remains an urgent challenge, a few surface markers, such as CD106, CD146, CD271, NG2, LEPR, are available for selecting distinct MSC subsets that exhibit different biological behaviours.^19^, ^20^, ^21^, ^22^, ^23^ Among these markers, VCAM‐1 (CD106) has been well documented that the biological function of MSC subsets with or without CD106 expression varies greatly. Concretely, in contrast to the CD106^−^ MSC subsets, CD106^+^ MSCs presented increased proangiogenic potential, preferable immunomodulatory properties and enhanced homing capacity, which collectively indicated the application prospect in cellular therapeutics.^19^, ^24^, ^25^

Particular functional MSC subpopulation markers have become a research hotspot, but there is no report on obtaining high‐quality specific MSC subpopulations in vitro. In the process of MSCs culture and passage in vitro, the competition and balance between different MSC subpopulations may change, which will lead to the decline or even loss of the proportion of some subgroup, such as CD34,^26^ and finally result in the alteration of cell function and therapeutic effects in clinical research.^27^, ^28^, ^29^ Therefore, it is vital to evaluate the cell quality of MSC subsets to ensure safe and efficient clinical treatment. When studying the function of MSC subpopulations, cell viability, apoptosis, proliferation and marker stability are the essential factors to be considered in the quality assessment of MSC subpopulations.^27^, ^30^, ^31^, ^32^, ^33^, ^34^ In other words, on the premise of ensuring cell quality, studying and exploring the functional advantages of a definite MSC subgroup can guarantee the stability and reliability of cell function to a great extent.

Here, we used CD106^+^ subpopulations as an example to conduct many key experiments on obtaining and validating clinical‐grade hUC‐MSC subgroups, including primary preparation, pre‐treatment, antibody incubation, flow sorting, quality and function test. In previous work, we have established a platform and methods for preparing clinical‐grade hUC‐MSCs.^4^, ^5^ On the premise of assuring the quality of initial hUC‐MSCs, we firstly focused on the pre‐treatment scheme by screening the most feasible existing approaches, then further explored the optimal antibody incubation conditions and adopted the most advanced flow sorting technology. Besides, cell quality (viability, apoptosis, proliferation and marker stability) and cell function (adhesion ability, migration ability, tubule formation ability, immunoregulatory function and wound healing and proangiogenic activity) of MSC‐CD106^+^ subgroups in two crucial clinical application scenarios, that were direct injection after cell sorting and post‐culture injection into bodies, were comprehensively assessed. We first formed a set of optimized operation systems to gain high‐quality clinical‐grade cell subpopulations from hUC‐MSCs based on specific markers. On this basis, we further established a five‐step operation procedure: *Step I* is the preparation of clinical‐grade MSCs. The pre‐treatment scheme we focused on is *Step II*, removing non‐specific sites and poor‐quality cells without cellular damage. *Step III* is to incubate the obtained cells with antibodies against specific functional markers under the optimal conditions. *Step IV* uses the most advanced flow sorting technology to obtain hUC‐MSC subpopulations with specific markers. Finally, *Step V* tests the quality of hUC‐MSC subpopulations with specific markers.

In a word, the optimized five‐step procedures to obtain clinical‐grade specific cell subpopulations from hUC‐MSCs cannot only improve the acquisition efficiency and the credibility of preclinical research but also provide valuable methodological guidance for accelerating the clinical transformation of specific hUC‐MSCs subpopulations.

## 2 RESULTS

### 2.1 MSC‐CD106 ^+^ subpopulations were obtained from clinical‐grade hUC‐MSCs

#### 2.1.1 Cultivation, expansion and identification of clinical‐grade hUC‐MSCs

In previous work, our group has established a Cell Factory and Biobank that is currently producing clinical‐grade hUC‐MSCs, which fully comply with the current Good Manufacturing Practice (cGMP) guidelines and meet the quality standards of the National Institute of Food and Drug Control (NIFDC)^4^, ^5^ (Figure S1). When the cells grew to 80%–85%, the cells were digested and prepared into a single‐cell state required for subsequent experiments. The generation time of hUC‐MSCs used in this study is strictly limited from generation 3 to 5.

#### 2.1.2 Optimized pre‐treatment scheme clinical‐grade hUC‐MSCs

**FIGURE 1:**
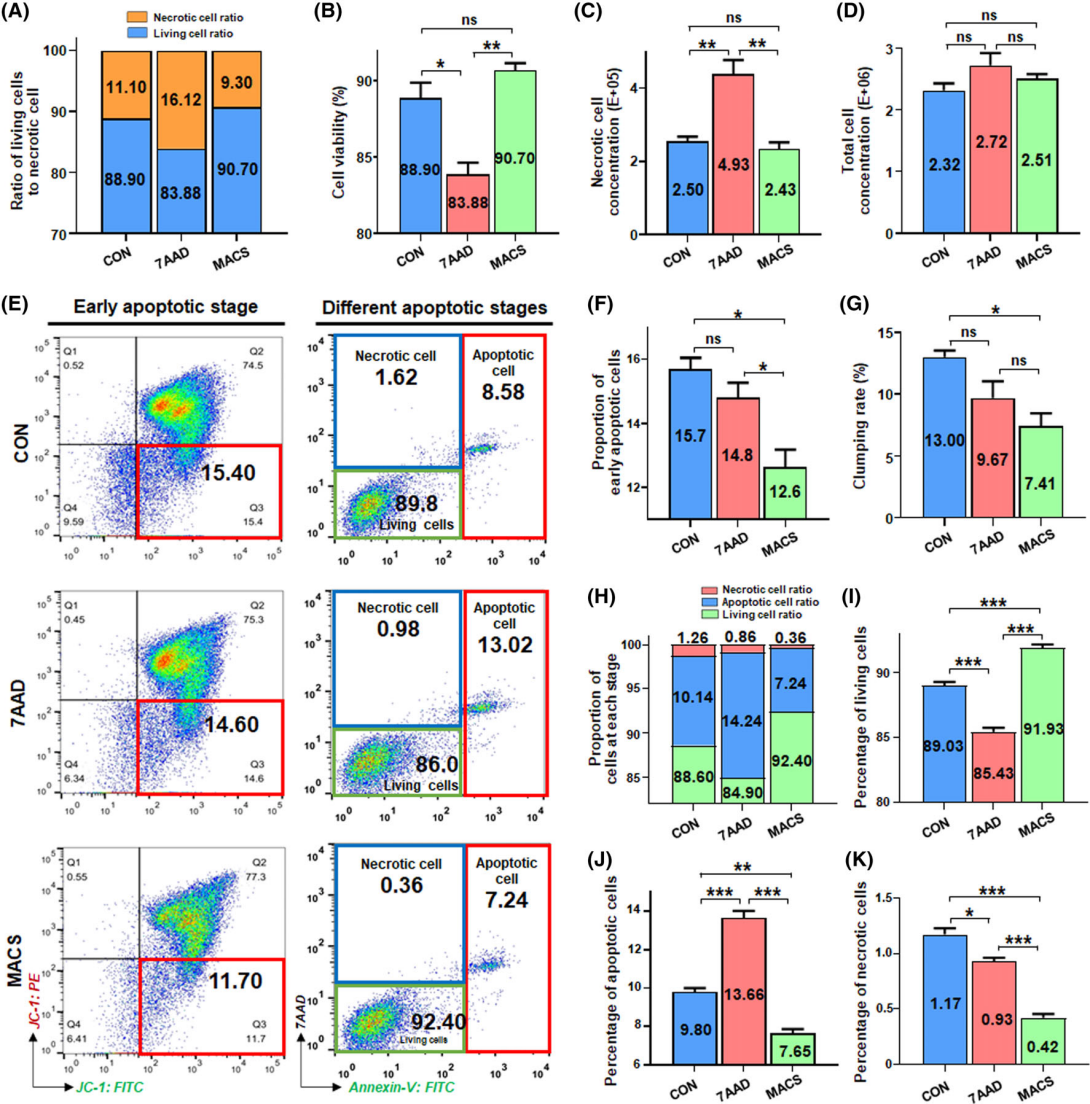
Direct assessment for cell viability and apoptosis state of the CD106^+^ hUC‐MSCs derived from the three groups after flow sorting. (A) Overview of living cells and necrotic cells of three groups after flow sorting. (B) Direct detection of cell viability of three groups after flow sorting. Then, detect necrotic cell concentration (C) and total cell concentration (D) of three groups and calculate clumping rate (G) based on the diameter of MSCs. (E) JC‐1, an ideal fluorescent probe, was used to directly detect MMP of three groups of cells and quantify the proportion of early apoptotic cells (left); annexin V combined with chemical dye 7AAD was used to detect cells in different apoptotic states in the three groups (right). (F) by directly detecting the changes of JC‐1 fluorescence value in three treatment groups. Furthermore, cell apoptosis of the CD106^+^ hUC‐MSCs derived from the three groups after flow sorting. (H) Overview of the proportion of cells at each stage, including living cells, apoptotic cells and necrotic cells. (I) Direct assessment of the percentage of living cells, percentage of apoptotic cells (J), and percentage of necrotic cells (K) according to the total number of cells (*n* = 3/group; all data shown as mean ± SEM, *p < 0.05, **p < 0.01, ***p < 0.001)

**FIGURE 2:**
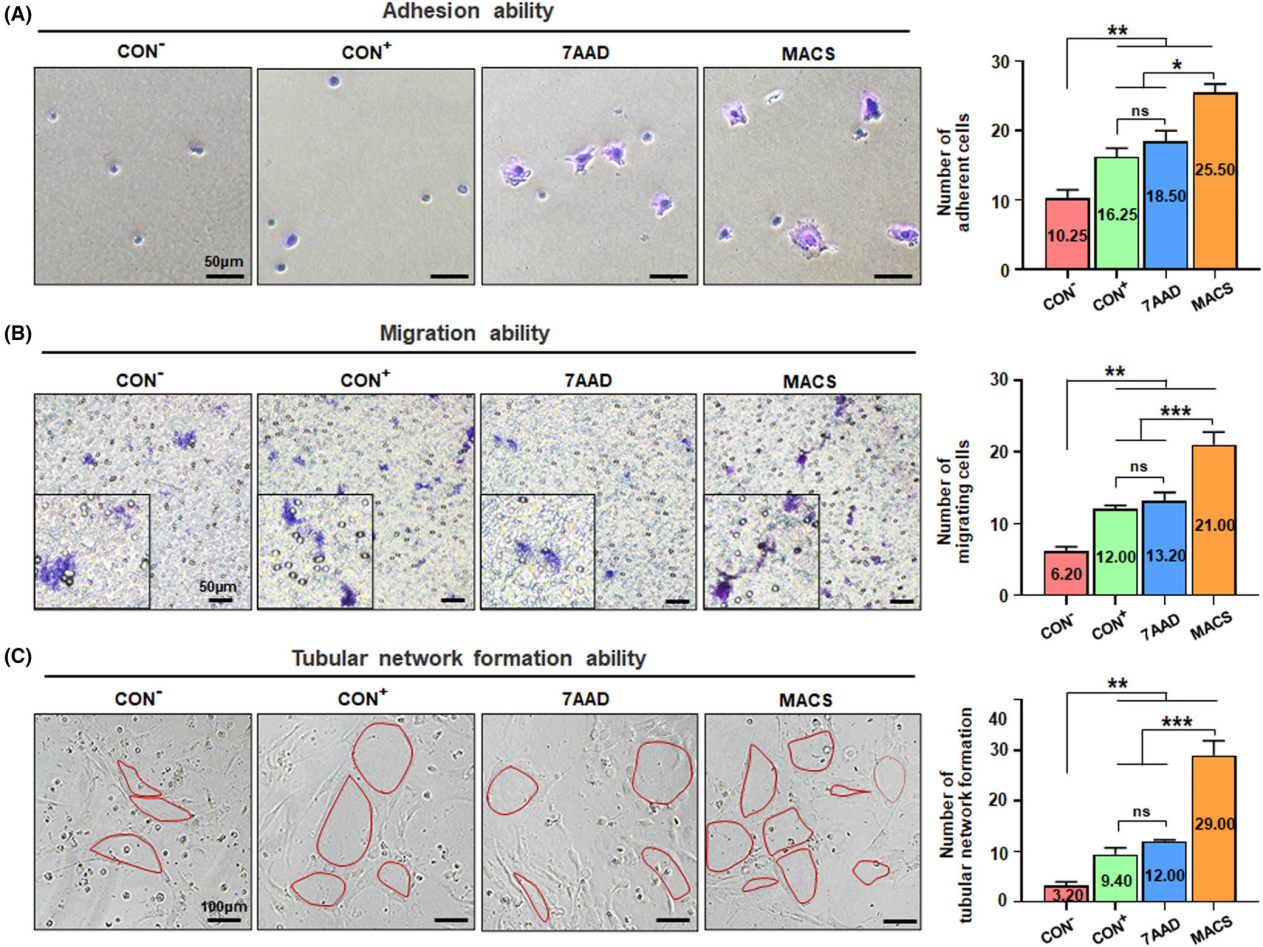
Direct assessment for the in vitro function of CD106^+^ hUC‐MSCs treated by the three methods after flow sorting. (A) Crystal violet staining was used to observe the number of adherent cells in the CON^−^ group of CD106^−^ hUC‐MSCs, and the three groups of CD106^+^ hUC‐MSCs treated by the three methods. Moreover, the number of unduplicated visual field statistics was randomly selected. (B) Four groups of cells were seeded directly with a transwell insert and counted the number of cells in the lower layer after 24 h to observe the migration function. (C) Four groups of cells were directly seeded on the matrigel‐coated 96‐well plate for 24 h and counted the microtubules (*n* = 3/group; all data shown as mean ± SEM, *p < 0.05, **p < 0.01, ***p < 0.001)

**FIGURE 3:**
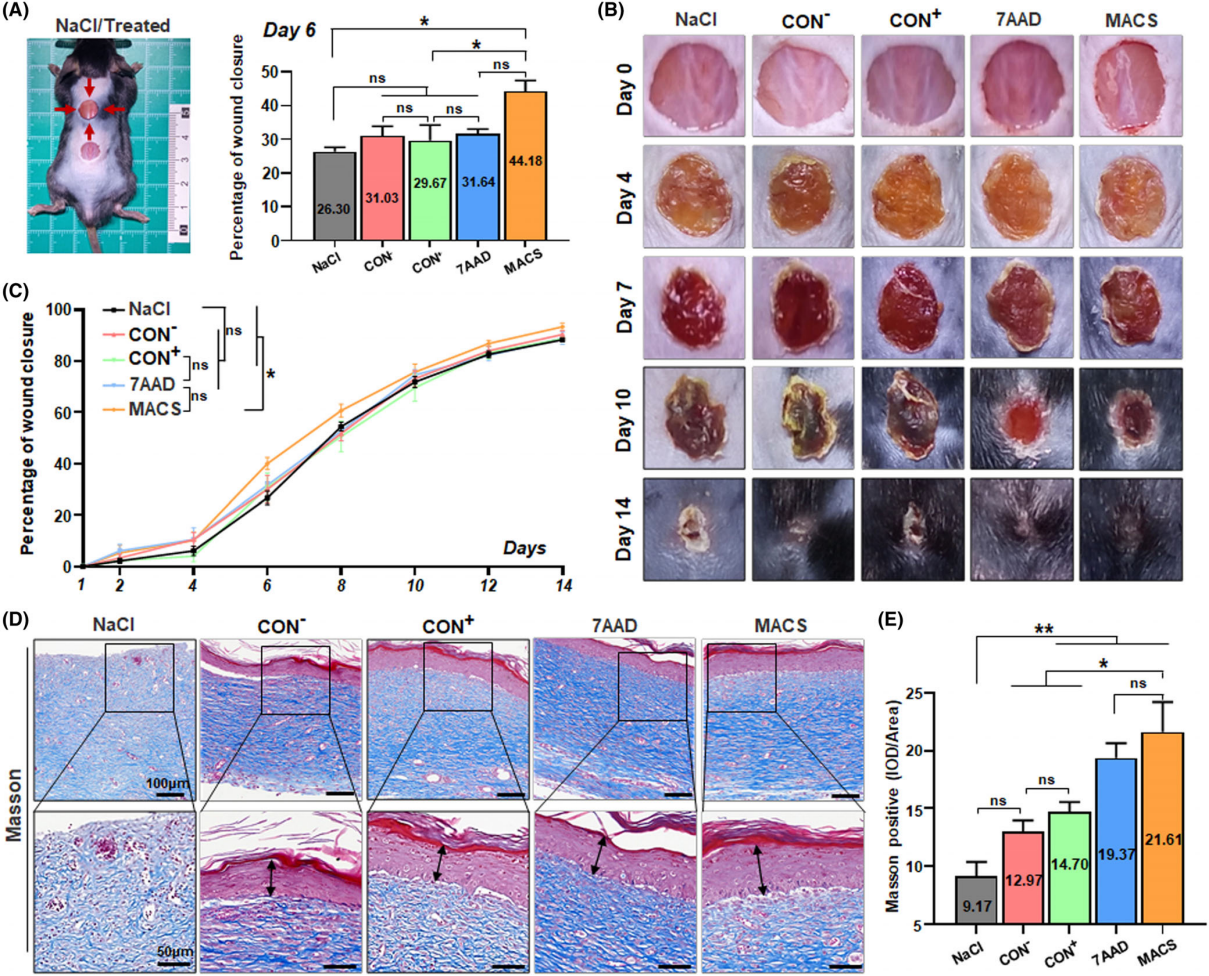
Direct assessment for the wound healing ability of CD106^+^ hUC‐MSCs treated by the three methods after flow sorting. (A) Wound model on C57 mice in the topical injection model. NaCl, CD106^−^ hUC‐MSCs, or CD106^+^ hUC‐MSCs treated by the three methods after flow sorting were injected directly 3–5 mm around the wound. And the statistical chart of the percentage of wound closure on Day 6 was represented as the intermediate time point. (B) The wound morphology and size were documented with a digital camera on Days 0, 4, 7, 10, and 14 after cell grafting. During observation, the mice were anaesthetized and placed flat with wound area and camera on a tripod in fixed positions, using identical light sources throughout the photo‐documentation. (C) The wound size and wound closure were measured semi‐quantitatively to analyse the percentage of wound closure according to the photo files taken every day (*n* = 6/group). (D) Masson trichrome staining of sections of the wound edges from different groups of mice at 14 days after wounding. Then, quantification of the Masson‐positive area was performed using Image J (*n* = 5/group; all data shown as mean ± SEM, *p < 0.05, **p < 0.01)

**FIGURE 4:**
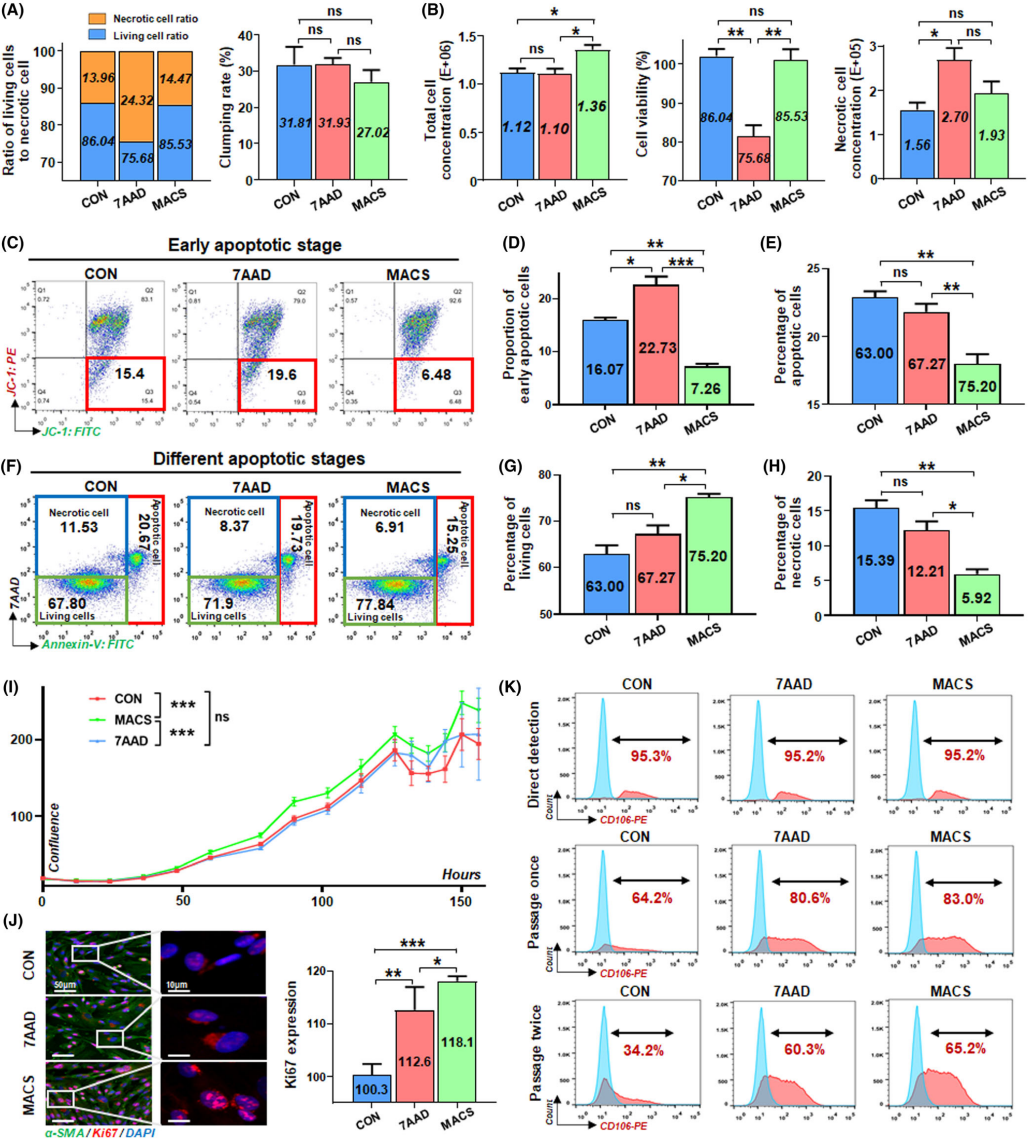
Assessment for cell quality of the CD106^+^ subgroup after cell culture. (A) Overview of living cells and clumping rate of three groups after incubation. (B) Detection of cell viability, necrotic cell concentration and total cell concentration in three groups after incubation. (C) JC‐1, an ideal fluorescent probe, was used alone to detect MMP of three groups of cells after incubation directly. (D) The changes in JC‐1 fluorescence value indicated the proportion of early apoptotic cells in three groups after incubation. (F) Annexin V and chemical dye 7AAD were jointly used to detect cells in different apoptotic states in the three groups after incubation. Assessment of percentage of living cells (G), apoptotic cells (E) and necrotic cells (H) (*n* = 3/group). (I) Three treatment groups were seeded and observed using a living cell analyser in real‐time. According to the images taken per 12 h, calculate the cell confluence and draw a curve, reflecting the cells' dynamic growth state (*n* = 8/group). (J) Immunohistochemical representative images showed the expressions of Ki67 in the three CD106^+^ subgroups adherent culture (scale bar = 50 μm). Squares in higher‐magnification inserts indicate the positive protein cells with several cells (scale bar = 10 μm). Then, quantification of the average fluorescence intensity of Ki67 was performed using Image J software (6 fields of view/group; all data shown as mean ± SEM, *p < 0.05, **p < 0.01, ***p < 0.001). (K) The stability of surface marker CD106 in the CD106^+^ subpopulation at different passages: zero, once and twice

**FIGURE 5:**
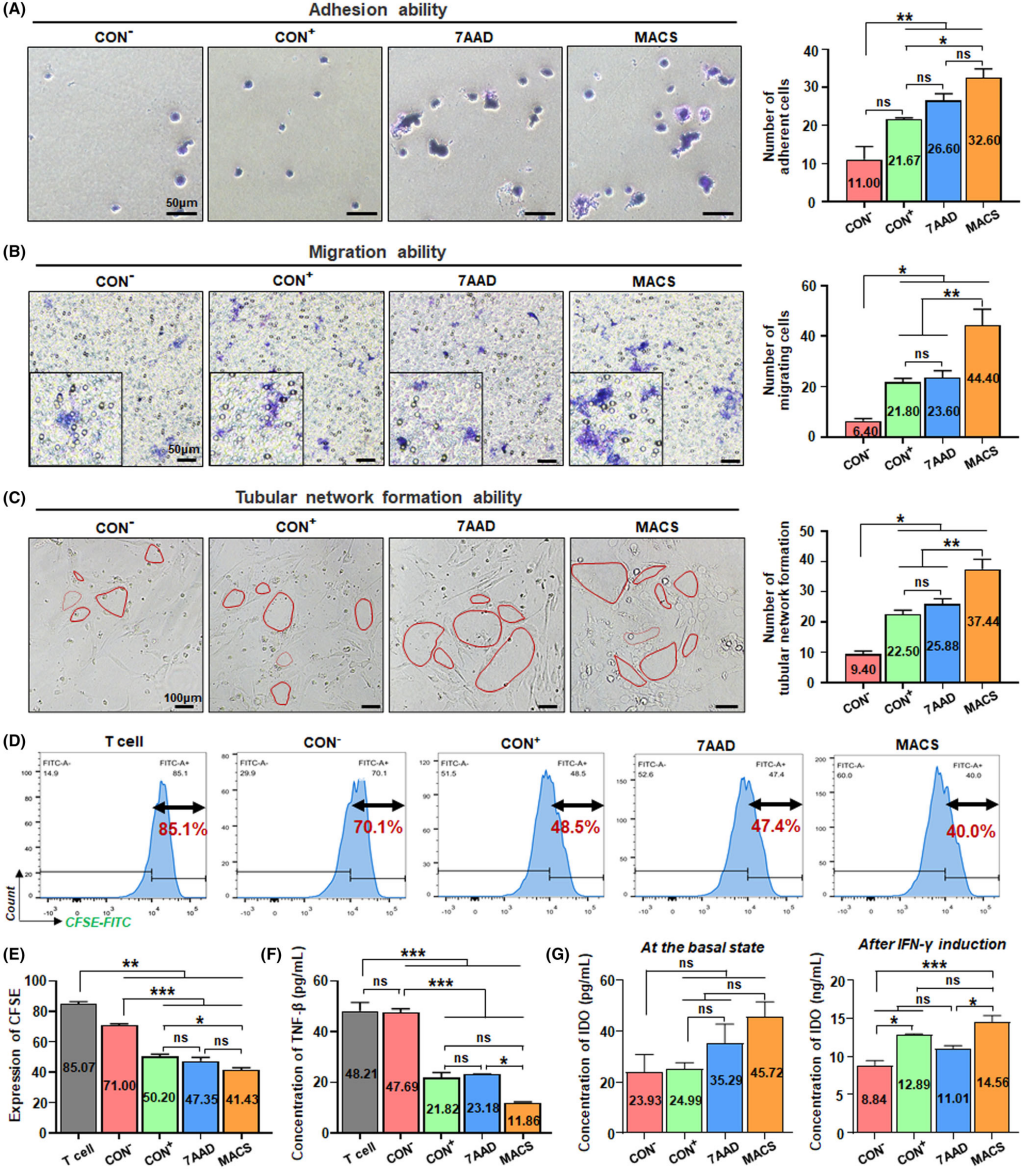
Assessment for the in vitro function of CD106^+^ hUC‐MSCs treated by the three methods after cell culture. (A) Four groups were cultured for 16 h and stained with crystal violet for 30 min to observe the number of adherent cells. Moreover, the number of unduplicated visual field statistics was randomly selected. (B) Four groups of cells after 16 h were seeded with a transwell insert, and counted the number of cells in the lower layer after 24 h to observe the migration function. (C) After 16 h of incubation, four groups of cells were inoculated on the matrigel‐coated 96‐well plate for 24 h and counted the microtubules. (D) CD3^+^ T‐cells incubated with CFSE dye were co‐cultured with different groups of cells to observe their ability to inhibit proliferation. And the positive expression rate of CFSE was analysed (*n* = 3/group). (E) ELISA detected TNF‐β concentration in the different groups of CD3^+^ T‐cells co‐cultured with CD106^+^ or CD106^−^ cells (pg/ml) (*n* = 3/group). (G) ELISA detected IDO concentration in the four groups of cells themselves (pg/ml) and IFN‐γ induced cells (ng/ml) (*n* = 4/group; all data shown as mean ± SEM, *p < 0.05, **p < 0.01, ***p < 0.001)

**FIGURE 6:**
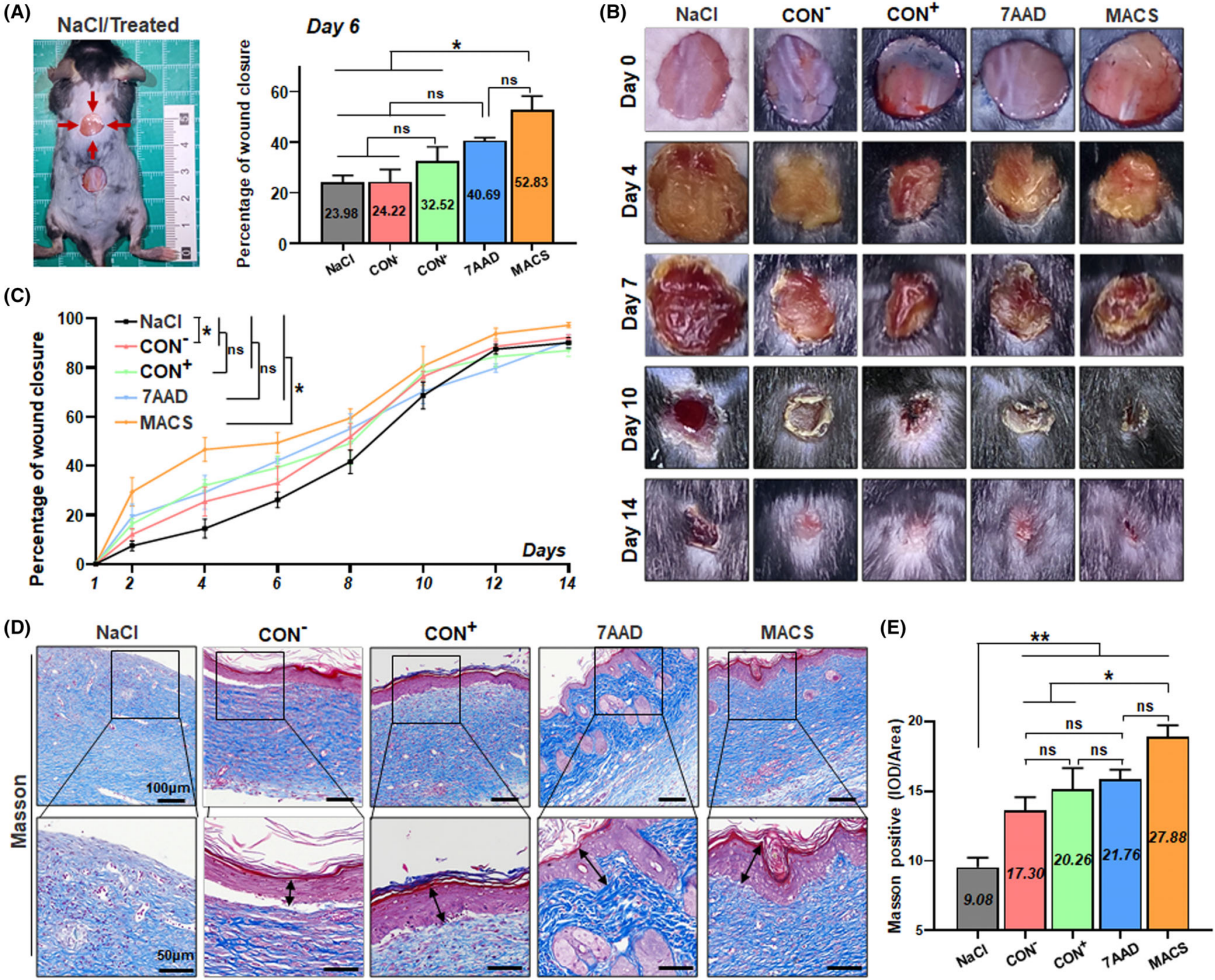
Assessment for wound healing ability of CD106^+^ hUC‐MSCs treated by the three methods after cell culture. (A) After incubation, NaCl, CD106^−^ hUC‐MSCs, or three groups of CD106^+^ hUC‐MSCs were injected 3–5 mm around the wound, and the statistical chart of the percentage of wound closure on Day 6 was represented as the intermediate time point. (B) Images of wounds photographed at Days 0, 4, 7, 10 and 14 post‐wounding in wound healing model. (C) The wound size and wound closure were measured semi‐quantitatively to analyse the percentage of wound closure (*n* = 3‐6/group). (D) Masson trichrome staining of sections of the wound edges after wounding and quantification of the Masson‐positive area was performed (n = 3–5/group; all data shown as mean ± SEM, *p < 0.05, **p < 0.01)

**FIGURE 7:**
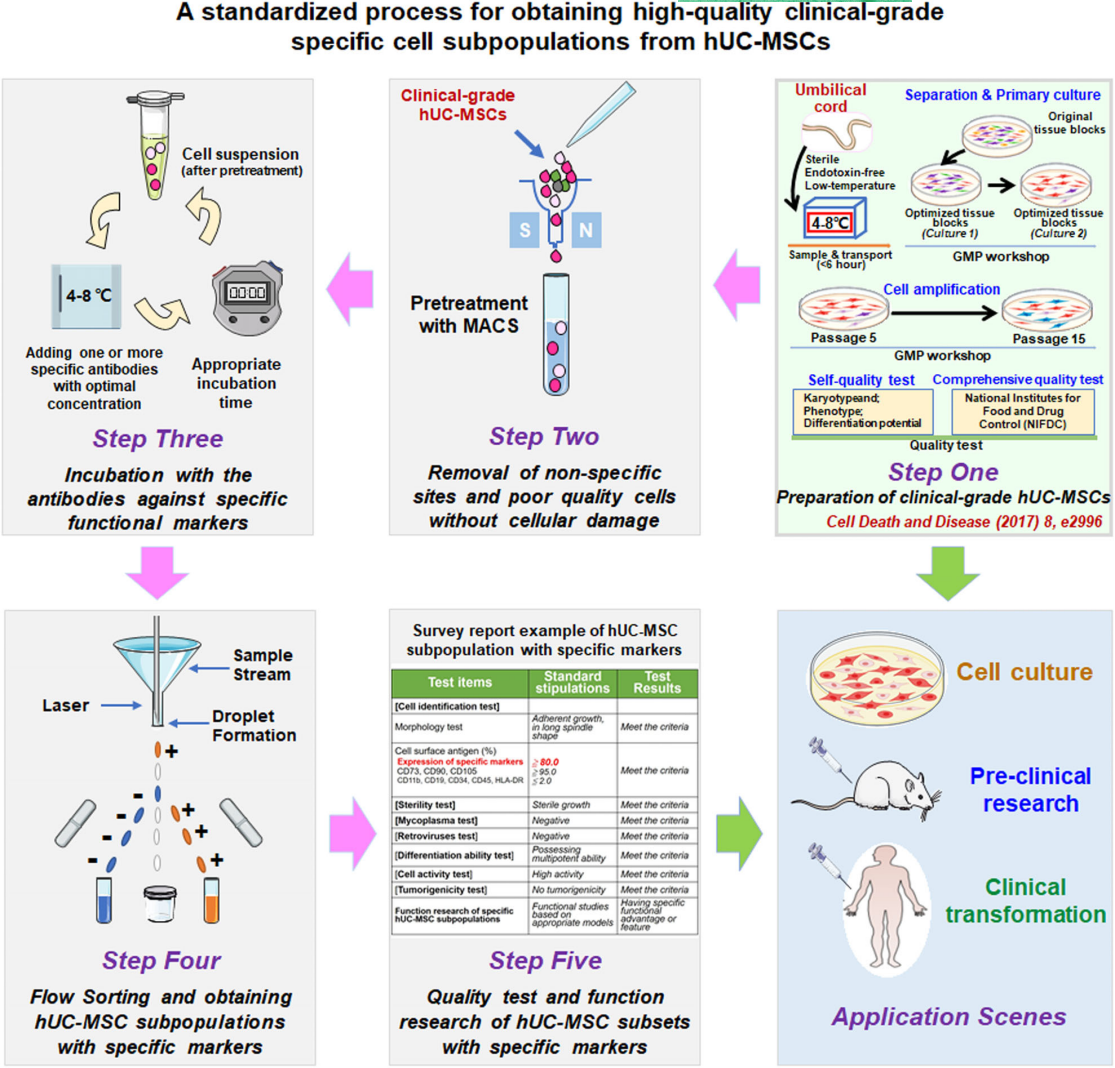
A general operation process was established for obtaining high‐quality specific subpopulations from clinical‐grade hUC‐MSCs. We established an optimized operation process for obtaining hUC‐MSC subpopulations with specific markers, which is also suitable for MSCs derived from other tissues. *Step One*. Preparation of clinical‐grade hUC‐MSCs. The clinical‐grade hUC‐MSCs obtained fully comply with the cGMP guidelines and meet the quality standards of the NIFDC. *Step Two*. Removal of non‐specific sites and poor‐quality cells without cellular damage. Treatment with MACS was proved as a safe and effective pre‐treatment by verifying multiple core indicators related to subpopulation quality. *Step Three*. Incubation with the antibodies against specific functional markers. Select the optimum condition of specific antibodies to ensure the purity of subsets obtained by subsequent sorting. *Step Four*. Flow Sorting and obtaining hUC‐MSC subpopulations with specific markers. This step has higher technical requirements related to flow cytometry. *Step Five*. Quality test and function research of hUC‐MSC subpopulations with specific markers. Do more research and verification to ensure the efficiency of clinical transformation of the obtained MSC subpopulations. *Application Scenes*. The clinical‐grade MSC subpopulations obtained through the above‐optimized operation process not only guarantee the quality of the cells in basic research and preclinical research but also provide safety assurance for clinical application

Before cell sorting, effective pre‐treatment is necessary, by which low‐quality cells and non‐specific sites can be removed to improve the efficiency of cell therapy. Several approaches have been developed to remove apoptotic and necrotic cells (Table S1). For clinical applications, safety is the primary consideration when selecting methods and reagents, while maximizing cell viability and state. The typical approach to remove late apoptotic and necrotic cells is using nucleic acid dyes, such as 4′,6‐diamidino‐2‐phenylindole 4 (DAPI), propidium iodide (PI)^35^, ^36^ and 7‐aminoactinomycin D (7AAD),^37^ to label double‐stranded DNA. Compared with 7AAD and PI, DAPI has stronger membrane permeability, so it is not suitable for clinical application of cells. A 7AAD is frequently used for multicolour fluorescence analysis, as its emission spectrum is narrower than that of PI, has less interference with other detection channels, and occupies fewer fluorescent channels.^38^ In addition to the above dye method, Dead Cell Removal MicroBeads of magnetic‐activated cell sorting (MACS) can physically remove dead cells, which can identify not only early apoptotic cells but also remove late apoptotic and necrotic cells. Therefore, among the four approaches above, 7AAD and MACS may be used as candidate cell pre‐treatment methods, but it is not clear whether they are different or better than the untreated group for specific cell subsets obtained later. To further clarify a better pre‐treatment scheme, we set up three groups: untreated, 7AAD‐treated and MACS‐treated groups before cell subpopulation sorting. Detailed grouping information is shown in Table 1. Through a comprehensive evaluation of the quality and function of the obtained MSC subpopulations, the MACS‐based pre‐treatment scheme was finally determined to be optimal (Table 2; Figure 1, 2, 3, 4, 5, 6, 7).

**TABLE 1:** Detailed grouping information and using CD106^+^ subpopulations as an example to optimize antibody incubation process

• CON (or CON^+^) group (hUC‐MSCs incubated with antibody without any pre‐treatment)
a. 10^6^ clinical‐grade hUC‐MSCs were resuspended with 1 ml physiological saline.
b. Add the CD106 antibody to cells following the recommended volume per test (1 Test).
c. After incubating the cells in the dark of 4°C for 30 min, wash and collect the cells.
• 7AAD‐treated group (hUC‐MSCs with 7AAD after antibody incubation)
a. hUC‐MSCs operate the same as the CON group.
b. Then 1 μM 7AAD was added to the cells, incubated at room temperature for 15 min in the dark.
c. Finally, cells were washed and collected for subsequent experiments.
• MACS‐treated group (hUC‐MSCs using dead cell removal microbeads of magnetic‐activated cell sorting).
a. Pass hUC‐MSCs through 100 μm nylon mesh and centrifuge hUC‐MSCs suspension at 300 × *g* for 10 min.
b. Resuspend cells in 100 μl of dead cell removal microbeads per 10^7^ total cells.
c. Mix well and incubate for 15 min at room temperature (20°C–25°C).
d. Place the column in the magnetic field of a suitable MACS Separator, and rinse the column with 1 × binding buffer.
e. Apply cell suspension onto the column, wash the column with 1 × binding buffer. Collect flow‐through containing live cells as subsequent experiments cells.
f. Finally, the CD106 antibody with 1 Test was added to every 1 × 10^6^ cells. After incubation, the cells were washed and collected.

*Note*: CON^−^ represents CD106^−^ subpopulations isolated from primary cultured human umbilical cord mesenchymal stem cells (hUC‐MSCs) for functional assays in vitro and in vivo.

**TABLE 2:** Assessment for cell quality and function of the CD106^+^ hUC‐MSC subpopulation derived from the three treatment groups

Clinical application Scenario 1: Direct injection after cell sorting								
Test index	Cell quality				Cell function			
Group	Safety	Viability	Apoptosis	Marker expression	Adhesion ability	Migration ability	Tubule formation ability	Wound healing ability
CON^+^	++	++	++	>90%	+	+	+	+
7AAD	/	+	+++	>90%	+++	+	+	+
MACS	++	+++	+	>90%	+++	++	++	++

Clinical application Scenario 2: Post‐culture injection into bodies											
Test index	Cell quality					Cell function					
Group	Cell count	Viability	Apoptosis	Proliferation	Marker stability	Adhesion ability	Migration ability	Tubule formation ability	Wound healing ability	Immuno‐modulatory function	Angiogenesis ability
CON^+^	+	++	++	+	35%–65%	+	+	+	+	+	+
7AAD	+	+	+++	++	60%–80%	+	+	++	+	+	+
MACS	+++	+++	+	+++	65%–85%	+++	++	+++	++	++	++

*Note*: “+” represents the value or degree of the test index. +, lowest; ++, moderate; +++: highest.

#### 2.1.3 Optimized antibody incubation process

Here, we used CD106^+^ subpopulations as an example to optimize antibody incubation process on obtaining and validating clinical‐grade hUC‐MSC subgroups. In order to guarantee the effective combination of antigen and antibody, we explored the antibody concentration and incubation time and finally formed the most suitable incubation conditions for the CD106 antibody. The results showed that the dose of 1 test (20 μl for each 1 × 10^6^ cells) and the incubation time of 30 min were the most suitable condition in our culture system (partial data were shown in Figure S2A, B). The antibody incubation procedures of the three groups were shown in Table 1. It should be noted that the recommended dose of different commercial antibodies is only a reference, and the optimal dose and incubation time must be strictly screened.

#### 2.1.4 Flow sorting of MSC‐CD106 ^+^ subpopulations

In the process of sorting hUC‐MSCs based on marker CD106, we select the most appropriate sorting conditions, such as pressure, threshold, nozzle (Figure S2C). Furthermore, when sorting specific cell subpopulations, it is worth noting that the threshold gate setting is one of the core parameters for distinguishing the expression of specific markers, which is particularly critical in flow sorting. We set the flow sorting threshold gate to about 10% distance from the positive expression rate to ensure the purity of the sorted CD106^+^ subpopulations (Figure S2D). The CD106^+^ subpopulations obtained from the three groups were named CON (or CON^+^), 7AAD and MACS, respectively. Correspondingly, CD106^−^ subpopulations, named CON^−^, were isolated from primary hUC‐MSCs and used for functional experiments in vitro and in vivo. In conclusion, we used the most advanced fluorescence‐activated cell sorting (FACS) technology to obtain the hUC‐MSC‐CD106^+^ subpopulations.

#### 2.1.5 Quality evaluation and functional verification of CD106 ^+^ subpopulations

In any clinical application scenario, the quality and corresponding functions are essential for efficacy. Therefore, for two clinical application scenarios: direct injection of MSC subgroups after cell sorting and post‐culture injection into bodies. Furthermore, by studying the effects of the three treatment groups, we established the indicators for comprehensive evaluation of the quality and function of the sorted CD106^+^ subpopulations, including cell viability, apoptosis, proliferation, marker stability, and in vitro abilities of adhesion, migration, tubule formation and immunoregulatory, and in vivo wound healing ability and proangiogenic activity (Table 2). Results 2.2–2.5 below were detailed quality and functional study results.

### 2.2 Direct assessment for cell quality of the CD106 ^+^ hUC‐MSCs derived from the three groups after flow sorting

In order to evaluate the feasibility of the clinical application scenario of ‘direct infusion after cell sorting’ of CD106^+^ subpopulations, we first performed a cell viability test, which is the most commonly used detection indicator to evaluate cell quality.^30^, ^39^ We stained the cells with the cell‐permeable dye acridine orange (AO) and PI impermeable to intact cellular membranes to label live cells and necrotic cells in the three groups, respectively (Figure S3).^40^ Among the three groups, the cell viability of the MACS group was the highest, while that of the 7AAD group was the lowest and the highest proportion of necrotic cells (Figure 1B, C). We speculate that 7AAD, as a chemical dye, may affect cells to some extent, while the process of removing non‐specific ectopic sites in the MACS group is a reversible antigen–antibody reaction (Table S1), which will not cause any potential damage to the cells themselves.

In addition to cell viability, apoptosis is also an important quality indicator. Cells at different apoptotic stages will show their typical biological changes.^41^ The three recognized characteristics are the decrease of mitochondrial membrane potential (MMP) in the early stage of apoptosis, the eversion of membrane phosphatidylserine (PS) in the early or middle stage, and the condensation and rupture of the nucleus in the late stage of apoptosis.^42^, ^43^, ^44^ Based on the above biological changes of apoptotic cells, we used fluorescent probes JC‐1 alone, or annexin V combined with chemical dye 7AAD to detect cells in different apoptotic states in the three groups (Figure S4). The fluorescence intensity of fluorescent probe JC‐1 is often used to define the proportion of cells from survival to death, that is, the decrease of MMP.^45^ The lowest value of MMP in the MACS group was the highest in the CON group (Figure 1E, F). Because annexin V cannot distinguish between necrotic and apoptotic cells and 7AAD dye cannot penetrate the complete cell membrane,^38^ annexin V is usually used in combination with 7AAD to improve the sensitivity and specificity of apoptosis detection.^31^, ^32^, ^33^ Flow cytometric analysis showed that the MACS group had the highest proportion of living cells, as well as the lowest proportion of apoptotic and necrotic cells; while the CON group had the highest number of necrotic cells, and the 7AAD group had the highest number of apoptotic cells (Figure 1G‐J). To sum up, the MACS group presented distinct advantages regarding reducing the apoptotic cells, increasing cell viability and ameliorating cell quality in the application scenario of direct clinical infusion (Figure 1).

### 2.3 Direct assessment for the cell function of CD106 ^+^ hUC‐MSCs treated by the three methods after flow sorting

After verifying the cell quality, it is necessary to ensure that the obtained CD106^+^ subgroups have the best function. CD106 is known to be an endothelial cell marker, mediates the intercellular interaction between MSCs and cardiac microvascular endothelium and its MSCs subgroup is one of the most important cellular components in the niche of bone marrow stem cells^46^, ^47^; more importantly, CD106^+^ MSCs presented enhanced homing capacity, increased proangiogenic potential, preferable immunomodulatory properties.^19^, ^24^, ^25^

For MSC replacement therapy, the adhesion ability of MSCs to vascular endothelial cells or tissue cells and extracellular matrix is the first step for them to play a role in repair and treatment.^46^ Using in vitro adhesion experiments, we found that CD106^+^ hUC‐MSCs in the MACS group presented the strongest adhesion ability, and especially significantly better than that of the CON^+^ group, while CD106^−^ hUC‐MSCs in the CON^−^ group presented the weakest adhesion ability (Figure 2A). In treating various refractory clinical diseases, the directional migration of MSCs in peripheral blood is also essential for its immune regulation and tissue repair function in the damaged tissue area.^48^ Our results showed that the MACS group has the highest migration capability of all groups (Figure 2B).

Proangiogenic activity is an advantaged property of CD106^+^ subgroups in hUC‐MSCs.^19^, ^24^ Thus, we conducted the tubular network formation assay in vitro and found the MACS group possessed obvious advantages in spontaneously forming intact tubular structures over other groups (Figure 2C). Thereafter, with the aid of the well‐established in vivo matrigel plug angiogenesis assay, we found that more macroscopic blood vessels were formed and distributed in the matrigel plugs in the MACS group (Figure S6).

Furthermore, we directly assessed the repair ability of the CD106^+^ hUC‐MSCs treated by the three methods after flow sorting using the mouse wound healing model,^49^ with a cell subcutaneous grafting procedure was performed around the full‐thickness wound (Figure 3A). The wound morphology and size were documented for 14 consecutive days, then the percentage of wound closure and collagen deposition were analyzed to evaluate which group had a positive effect on wound healing. The results showed that the wound healing process of MACS group had better repair ability than the other groups, and was significantly superior to the CON^+^ group (Figure 3B–D).

Collectively, the above results suggested that CD106^+^ cells in the MACS group presented the best cell quality and corresponding cellular functions after flow sorting. The corresponding subgroup acquisition method may be the optimal treatment for the scene of direct clinical application after sorting.

### 2.4 Assessment for cell quality of the CD106 ^+^ subgroup after cell culture

The number of MSC subpopulations based on different surface markers is different, and for subpopulations with a small proportion, it may take a certain amount of culture time after sorting to amplify to the right number for clinical infusion. In order to evaluate the practicability of using cells in ‘post‐culture injection into bodies’ in clinical application scenarios, we initially select 16 h as the candidate adhesion culture time, which cannot only ensure the recovery of cell adhesion state but also prevent excessive cell expansion. Then, the same initial number of three groups of CD106^+^ subgroups were selected for adherent culture without changing the medium. The results showed that the total cell concentration and viability of the MACS group were the best, while the cell viability of the 7AAD group was the lowest (Figure 4B). Similar to the experimental steps described in the previous application scenario, adherent cells and supernatants cultured for 16 h were collected and combined with fluorescent probes annexin V, MMP and dye 7AAD to evaluate apoptosis comprehensively (Figure 4C, F). The proportion of early apoptotic cells was the lowest in the MACS group and the highest in the 7AAD group (Figure 4C, D). Flow cytometric analysis showed that the MACS group had the highest proportion of living cells and the lowest proportion of apoptotic and necrotic cells, while the CON group had the highest number of necrotic and apoptotic cells (Figure 4E‐H).

Furthermore, we focused on two other factors that are equally influential to the quality of survival of subpopulations: cell proliferative ability and the stability of markers.^27^, ^28^, ^29^, ^34^, ^50^ CD106^+^ hUC‐MSCs from the three treatment groups were seeded at the same density and observed using a living cell analyzer in real‐time. According to the images taken at different time points (Video S1, and Figure S5), the cell confluence was calculated to reflect the cell proliferation function. Compared with the CON and 7AAD groups, the proliferation ability of the MACS group was the highest (Figure 4I). In addition, the expression of proliferation marker Ki67 in the MACS group was significantly higher than that in the other two groups (Figure 4J). For MSC subpopulations, the stability of particular markers is critical to their specific biological functions. The expression of CD106 of the three groups was detected at three‐time points: the cells were not passaged, the cells were passaged one time, and the cells were passaged two times. The positive rate of CD106 markers in the three groups decreased to varying degrees due to the expansion of cells. There was significant marker loss in the CON group, but the CD106 marker in the MACS group was more stable than the other two groups (Figure 4K). In general, after culture of CD106^+^ hUC‐MSCs, the MACS group was better than the other two groups and had prominent characteristics and advantages (Figure 4).

### 2.5 Assessment for the cell function of CD106 ^+^ hUC‐MSCs treated by the three methods after cell culture

To assess the cell function of CD106^+^ hUC‐MSCs treated by the three methods in the clinical application scenario of post‐culture injection, we first examined the in vitro function of cell adhesion, migration, tubule network formation and immunoregulation of the CD106^+^ cells treated by the three methods, using CD106^−^ cells as the negative control. The MACS group had a more distinct advantage than the other two groups in the above function as in the previous scenario of direct clinical application after sorting (Figure 5A‐C).

Besides, CD106^+^ subpopulations were specialized in immunosuppression.^19^ Therefore, the T‐cell proliferation inhibition assay was performed to detect the immunomodulatory ability of the CD106^+^ cells treated by the three methods, and the results showed that CD106^+^ cells in the MACS group had a more potent inhibition of CD3^+^ T cell proliferation than that in the CON^+^ group (Figure 5D‐F). Furthermore, the determination of intracellular cytokine contents on the CD3^+^ T cells of the co‐cultures showed that different groups decreased to some extent the expression of TNF‐β mainly expressed in CD3^+^ T cells,^51^ while the MACS group was more effective (Figure 5F). Moreover, potency assays were also performed to characterize the significant immunoregulation ability of hUC‐MSCs to further evaluate cell functionality at the basal state and pro‐inflammatory conditions. It is considered that IDO (indoleamine 2,3‐dioxygenase 1) is one of the essential active molecules mediating the immunomodulatory function of hUC‐MSCs,^52^ and detecting the activity of IDO secreted by hUC‐MSCs can effectively evaluate the biological effectiveness of the immunomodulatory properties of hUC‐MSCs.^53^, ^54^, ^55^ As a result, the CD106^+^ cells of the MACS group had certain advantages among the three groups, especially after IFN‐γ induction (Figure 5G).

Finally, we measured the wound size at different time points after cell injection at this scene again in the mouse wound healing model (Figure 6A, B). Compared with the NaCl group and CD106^−^ group, the CD106^+^ subpopulations significantly accelerated the wound healing in general, and the MACS group presented more effective skin healing (Figure 6C), and the corresponding collagen deposition in this group of mice was also higher (Figure 6D).

Collectively, these data revealed that the MACS group had multitudinous preponderances related to improving quality and optimizing function both in vitro and in vivo. Again, the pre‐treatment scheme may be an excellent method in post‐culture injection scenes.

### 2.6 An optimized operation process was established for obtaining high‐quality specific subpopulations from clinical‐grade hUC‐MSCs

Based on all the studies, including the above results, we explored and improved the critical processes of obtaining CD106^+^ hUC‐MSC subpopulations. The CD106^+^ subpopulations obtained through the optimized operation process have higher activity and stability. In a nutshell, the entire operation process consists of five key steps (Figure 7), as follows:

*Step I. Preparation of clinical‐grade hUC‐MSCs*. All of the technologies involved in this step have been completed in the early stage,^1^, ^2^, ^3^, ^5^, ^39^ and the clinical‐grade hUC‐MSCs were obtained entirely to comply with cGMP guidelines and meet the quality standards of NIFDC.

*Step II. Removal of non‐specific sites and poor‐quality cells without cellular damage*. In this step, we pre‐treated clinical‐grade hUC‐MSCs through various schemes and finally screened MACS treatment as a safe and effective pre‐treatment by verifying multiple core indicators related to subpopulation quality (Tables 2 and S1).

*Step III. Incubation with antibodies against specific functional markers*. In this study, we selected CD106 as an example, and the MACS‐based pre‐treated hUC‐MSCs from *Step II* were incubated with the CD106 antibody. It should be noted that the most suitable concentration and time of specific antibodies must be explored to ensure the purity of subsets obtained by subsequent sorting.

*Step IV. Flow Sorting and obtaining hUC‐MSC subpopulations with specific markers*. This step has higher technical requirements related to the flow cytometry, including the quality of flow cytometer, the level of personnel operation, the setting of sorting parameters and many other factors.

*Step V. Quality and function test of hUC‐MSC subpopulations with specific markers*. This step comprehensively evaluates the MSC subpopulations obtained above and reasonably select the test items according to the specific application scenarios. In addition to the cell quality and specific function tests focused in this study include cell identification test, sterility test, mycoplasma test, differentiation ability test, and tumorigenicity test (this part of the data is not shown).

## 3 DISCUSSION

MSCs have exerted therapeutic effects in various tissues and organs and achieved promising results in clinical trials.^56^, ^57^, ^58^, ^59^, ^60^, ^61^, ^62^, ^63^, ^64^, ^65^, ^66^ The clinical efficacy of MSCs depends mainly on cell quality. However, MSCs are a heterogeneous population, and the evolution of their characteristics and biological functions is still obscure.^14^, ^39^, ^67^ The functional heterogeneity of MSC‐based cytotherapy has attracted the attention of researchers in basic science and translation studies, suggesting an urgent need to distinguish between different types of MSC subpopulations.^13^, ^68^ Identifying specific cell subpopulations based on surface markers is currently the most important and feasible idea and method. However, even after elucidating the preclinical functions of a specific subpopulation, as far as we know, there are no reports of assessment methods currently available for obtaining high‐quality MSC subpopulations that can be used in clinical applications. This study selected CD106^+^ hUC‐MSC subgroups as a preliminary exploration model, which are relatively sufficient in multiple preclinical studies.^19^, ^24^, ^25^ After continuous optimization, we constituted the five core steps of standard operating procedures for obtaining high‐quality clinical‐grade hUC‐MSC subpopulations with specific markers (Figure 7).

Preparing and acquiring clinical‐grade hUC‐MSCs in *Step I* is an essential starting link. The quality of hUC‐MSCs may directly affect their functional results; however, the culture conditions of hUC‐MSCs from different sources and different laboratories may produce different experimental results. Thus, establishing a standardized procedure and ensuring its repeatability, safety and effectiveness are crucial for the clinical application of hUC‐MSCs subpopulations.^69^, ^70^, ^71^ As mentioned earlier, our group previously established a complete system for obtaining clinical‐grade hUC‐MSCs according to the cGMP guidelines, and the obtained cells fully meet the quality standards of the NIFDC^4^, ^5^ (Figure S1). Briefly speaking, *Step I* has laid a critical foundation for the acquisition, functional research and application of clinical‐grade specific cell subpopulations from hUC‐MSC.

Before specific antibody incubation, poor‐quality cells must be removed, such as early apoptotic, late apoptotic and necrotic cells produced during cell culture. It is to prevent antibodies from being combined with non‐specific sites during antibody incubation to avoid false‐positive results and reduce sorting purity, which will further affect their biological functions and cut down the efficiency of cell therapy. Therefore, it is necessary to pre‐treat the cells before entering the following process. Table S1 lists the typical approaches for removing poor‐quality cells, of which the most commonly used is the labelling of double‐stranded DNA with nuclear acid dyes. Among these dyes, using 7AAD instead of PI presents several advantages: identifying late apoptotic and necrotic cells, a narrower emission spectrum, less interference with other detection channels, and occupying fewer fluorescent channels.^38^ In addition to the dyes, MACS could recognize a moiety in the plasma membrane of apoptotic as well as dead cells, and even early apoptotic cells with an intact cellular membrane, which can effectively remove premature and late withering and dead cells. The safety and efficiency of immune‐MACS are widely accepted for sorting cells with specific markers.^72^ Nevertheless, MACS is only suitable for single‐marker sorting, and flow sorting is required when cells need multi‐fluorescence channel sorting.^73^ Thus, 7AAD and MACS may be used as candidate cell treatments before subgroup sorting; however, whether the cell quality obtained by the two treatments is different has not been reported.

Here, we focused on *Step II* of pre‐treatment scheme. By comparing the cell quality and the function of specific markers, we comprehensively evaluated the clinical transformation efficiency of CD106^+^ hUC‐MSCs from three groups (untreated groups, 7AAD‐treated and MACS‐treated) under two different application scenarios. All results were consistent to indicate that the cell subpopulations obtained after MACS pre‐treatment presented the best quality with multidimensional superiorities (Figures 1, 2, 3, 4, 5, 6, Table 2). Therefore, in *Step II*, we selected the optimized MASC pre‐treatment approach as one of the critical steps to obtain a subgroup process of high‐quality clinical‐grade hUC‐MSCs.

After proper pre‐treatment of clinical‐grade hUC‐MSCs, before sorting, there is a crucial step of flow sorting—— antibody incubation. In *Step III*, one or more antibodies with specific surface markers are added to the pre‐treated clinical‐grade hUC‐MSCs for antigen–antibody reaction under the optimum conditions. The main factors determining the specific immune response include the number of cells, antibody concentration, incubation time and ambient temperature. However, particular incubation conditions need to be explored for different antibodies to ensure antibodies' full and specific binding to cell surface antigens.

Followed by *Step IV*, the cells were obtained by flow sorting, which is a most general but fairly sophisticated and is still improving, involving the quality of the flow cytometer, the level of personnel operation, the setting of sorting parameters and many other factors. In a word, this study selects the most advanced FACS technology and combines the experience of multiple sortings, such as the threshold gate, to ensure the purity of subsets after sorting and the efficiency of cell therapy. It is worth mentioning that it is recommended to design and adopt specific quality control steps based on different surface markers of particular subpopulations.

A comprehensive assessment of the quality and function of the subpopulation is *Step V* and the optional step to optimize the procedures. Verifying cell quality and its corresponding function is crucial for application in any clinical treatment scenario. Therefore, this study selected two critical clinical application scenarios: direct injection after cell sorting and injection into the body after subpopulations culture. Relevant quality detection and corresponding functional detection of hUC‐MSCs subpopulations with specific markers comprehensively evaluate the cell efficacy for clinical transformation. Due to the need to enrich subpopulations based on specific markers, cell subpopulations must maintain high‐level expression of specific markers and remain stable to some extent to ensure the corresponding biological functions, which is also a significant factor for cell subpopulations to be used for research and transformation. Our experimental results show that the positive rate of the sorted subgroups in culture is gradually decreasing, which is consistent with previous reports.^28^ Therefore, due to the generation limitation in the clinical application of MSCs, it is suggested that the hUC‐MSCs subpopulations obtained by sorting should be passaged up to two times, and the positive rate of this marker should not be less than 80%. Although the initiating cells are already clinical‐grade, it is still vital to reconfirm MSC subpopulations' basic characteristics and corresponding safety indexes. Therefore, besides the cell quality and marker‐related functions in this study, other related experiments are needed, such as cell identification test, sterility test, mycoplasma test, differentiation ability test and tumorigenicity test. After testing, the above safety indexes of the sorting MSC subpopulations based on specific surface markers meet the standards. In general, the clinical‐grade MSC subpopulations obtained through the above five steps have higher activity and stability, which can supply assurance for cell quality in basic and preclinical research.

## 4 MATERIALS AND METHODS

### 4.1 Cultivation, expansion and identification of clinical‐grade hUC‐MSCs

The Ethics Committee approved all procedures involving human subjects in this study at the Third Affiliated Hospital of Sun Yat‐sen University (Approval number: 2017–19), and all the patients gave their written informed consent to participate. Clinical‐grade hUC‐MSCs were used in this study which was greatly optimized in previous research. hUC‐MSCs isolation and culture were performed according to a standard operating procedure (SOP) established in our lab as previously described.^4^, ^5^ The entire link, including isolation, cultivation, identification, quality control and storage, was confirmed to the quality standards. Clinical‐grade hUC‐MSCs were seeded at an initial density of 1 × 10^4^ cells cm^−2^ in 10 cm dishes, cultured for 24 h in a constant temperature incubator at 37°C, 5% CO_2_ and saturated humidity overnight.

### 4.2 Magnetic‐activated cell sorting to remove dead cells

Centrifuge cell suspension at 300 × *g* for 10 min. Resuspend cells with Dead Cell Removal MicroBeads (Miltenyi Biotec, Germany). Mix well and incubate for 15 min at room temperature. Add the cell suspension to the column. Rinse with 1× binding buffer and collect effluent as living cells. In the cell suspension, early apoptosis, late apoptosis and necrotic cells are magnetically labelled and retained within the column, and the living cells in good condition are passed through the separation column.

### 4.3 Incubation of cell surface marker antibodies and labelling with 7AAD dye

Add the CD106 antibody (BD, USA) to the cells obtained in cells that did not use the Dead Cell Removal Kit, incubate them in the dark at 4°C for 30 min, and wash them twice. Then, the cells were added 1 μM 7AAD (Elabscience, China), incubated at room temperature for 15 min in the dark and washed twice. And set up the untreated control group (CON or CON^+^), which only carried out CD106 antibody incubation.

### 4.4 Cell sorting and detection of cell surface markers

Perform flow sorting on untreated control group (CON or CON^+^), 7AAD dye labelling group (7AAD), and magnetic‐activated cell sorting group (MACS) by BD FACSAria II (Becton Dickinson, USA). At the same time, CD106^−^ subpopulations were sorted from the primary cultured hUC‐MSCs (CON^−^). After sorting, the three groups were analysed by Guava easyCyte Flow Cytometer (Luminex, China) to detect positive expression for judging whether the sorting is successful and whether the surface markers are stable.

### 4.5 Detection of cell viability

Centrifuge the three groups after sorting, and add AO and PI (Countstar, USA, D21011). AO is a fluorescent dye with membrane permeability that stains nuclear DNA and RNA. Through the FL1 channel, the cell nucleus is uniformly fluorescent in green or yellow–green, and the fluorescence is weakened or even disappears in the necrotic cells. PI is a DNA‐binding dye with no membrane permeability and cannot penetrate the membrane of living cells. It can only stain dead cells. It uses the FL2 channel to produce red fluorescence.^40^ Use a fluorescent cell analyzer (Countstar®, Countstar Rigel v3.5, China) to capture cell images and analyze cell viability and other indicators.

### 4.6 Detection of apoptosis

Resuspend the three groups after sorting with 1 × binding buffer, add Annexin V‐FITC (DOJINDO, Japan) fluorescent probe and 7AAD, incubate at room temperature for 15 min in the dark, and use Guava easyCyte Flow Cytometer (Luminex, China) to detect.

### 4.7 Detection of cell MMP

Centrifuge the three subgroups, add 10ug/ml JC‐1 (Invitrogen, USA), and incubate in a constant temperature incubator at 37°C for 30 min. After washing, use Guava easyCyte Flow Cytometer (Luminex, China) to detect.

### 4.8 Preconditioning of hUC‐MSCs and detection of IDO

1.9 × 10^5^ cells/ml three groups of cells were seeded to a 24‐well plate in 300 μl for 24 h, then treated with IFN‐γ (Pepro Tech, USA) with 10 ng/ml for 24 h. hUC‐MSCs were treated with IFN‐γ according to the ISCT® recommendations.^74^ Cell‐free supernatants were collected and kept in a refrigerator at −80°C. All IDO ELISA assay kits (Abcam, USA) were used following the supplier's instructions.

### 4.9 Detection of CD3 ^+^ T cells proliferation inhibition

T cells were established from normal adult PBMCs. Human CD3^+^ T cells were prepared separately by using separation beads (MACS cell isolation kit, Miltenyi Biotec) and stimulated with 20 μnits/mL IL‐2 added to culture medium for 72 h. These cells were more than 95% CD3‐positive. Then, 20 μg/ml mitomycin C was used to inhibit three groups of MSCs proliferation. Furthermore, CD3^+^ T cells were incubated at room temperature with 2 μmol/L CFSE dye for 20 min and washed twice with RPMI1640 containing serum. Finally, purified CD3^+^ T cells (3 × 10^5^ cells/well in 24‐well plates) from the PBMCs of healthy donors were co‐cultured with three groups of cells (3 × 10^4^ cells/well: effector/target ratio = 10:1). Moreover, use Guava easyCyte Flow Cytometer (Luminex, China) to detect the expression of CFSE. T cell activation was evaluated by measurement of human TNF‐β production by using ELISA (elabsciences, China).

### 4.10 Adhesion assays

Adhesion assays were performed using a 96‐well plate on which 2 × 10^4^ MSCs were plated. After incubation at 37°C for 30 min, wells were gently washed three times with PBS, and then the cells were stained with crystal violet.

### 4.11 Tubular network formation assay in vitro

Three groups of cells were seeded at 2 × 10^4^ cells/well gently on an matrigel‐coated (BD Biosciences, USA) 96‐well plate. Photographs were taken by Microscope (Olympus, USA) 12 h later. Tube numbers in each well were counted. Each sample was performed in triplicate.

### 4.12 Migration assays

Three group cells suspended in serum‐free media were added to a transwell insert (pore size 8 μm, Corning, USA) and placed on the 24‐well plate containing serum‐free medium containing 20 ng/ml vascular endothelial growth factor and incubated in a 37°C/CO_2_ incubator for 24 h. After incubation, cells in the transwell's upper chamber, which were not migrated, were removed gently with a cotton swab. Migrated cells were fixed with 4% paraformaldehyde for 30 min at room temperature and stained with gentian violet (Solarbio, China).

### 4.13 Wound healing assays and wound size and closure analysis

All animal experiments followed the Peking Union Medical College Animal Care and Use Committee guidelines. Using an 8 mm diameter punch biopsy tool, a full‐thickness wound was induced by punching through both sides of the fold of the dorsal skin, at this moment inflicting two identical bilateral wounds of the same size. C57BL/6 mice at the age of 6 weeks were divided into 4 equal groups stochastically. Each group was treated by different interventions of NaCl, MSC‐CON^−^, MSC‐CON^+^, MSC‐7AAD and MSC‐MACS, respectively. MSCs were cultivated and harvested by trypsinization and resuspended in NaCl with 1.5 × 10^6^ cells/ml; 3 × 10^5^ cells with 200 μl PBS were carefully injected into the surrounding skin 3–5 mm away from the wound of each mouse in the treatment group. Computer planimetry was used to measure the contracture degree and wound size every day for 2 weeks. The wound was exposed to the camera, maintaining the same distance, aperture, exposure and a ruler adjacent to the wound as the scale bar. With standardized documentation photos across the time points, the sizes of the healing wounds in both groups were analyzed using Image J analysis software. Both groups were sacrificed on Day 14, and the full‐thickness skin tissues were collected and stored in paraformaldehyde fix solution for Masson's trichrome staining to evaluate collagen deposition.

### 4.14 Matrigel plug angiogenesis assay in vivo

Six‐week‐old nude male mice were purchased from the Institute of Experimental Animal (Beijing, China); 1 × 10^6^ cells/ml different groups of cells were resuspended in 400 μl matrigel and implanted into the dorsal area of 6‐week female nude mice. Matrigel supplement with NaCl served as the negative control. Each group contained three to six mice. Twenty‐one days later, matrigel implants were harvested, photographed, fixed, sliced and stained with haematoxylin and eosin (H&E) staining to evaluate neovascularization. Micro‐vessels were photographed and calculated under the microscope.

### 4.15 Detection of cell proliferation ability and confluence

The three CD106^+^ subgroups after sorting, 8000 cells per well were seeded into 96‐well plates, and 8 wells were repeated in each group, placed in Counstar Rigel (Incucyte S3, USA), and photographed every 6 h. Cell confluence was calculated based on the captured images to generate dynamic growth.

### 4.16 Immunofluorescence staining

Cell cultures were pre‐incubated in 10% normal donkey serum, Triton 0.3% in PBS for 30 min. Cell cultures were incubated overnight at 4°C with the following antibodies: mouse monoclonal anti‐human‐α‐SMA (R&D, USA) and rabbit polyclonal anti‐Ki67 (proteintech, USA). Add secondary antibodies (Invitrogen, USA) after washing three times for 5 min each in PBS. Nuclei were visualized using 2 μM DAPI (Sigma, Germany). TissueFAXS (TissueGnostics GmbH, Vienna Austria) with a Zeiss Axio Imager Z2 Microscope System at ×20 magnification to acquire Immunofluorescence image.

### 4.17 Statistical analysis

All data were shown as means ± SEM. IDO and wound Size and Closure Analysis was analyzed using Image J software (version 1.8.0, National Institutes of Health, USA). Statistical analyses were performed with GraphPad Prism 8.0 software (GraphPad Software, USA) and expressed as mean plus or minus standard error of the mean. For comparisons of the mean between two groups, statistical analysis was performed unpaired two‐tailed Student's *t*‐test as indicated in the bar graph. Statistical significance was set at *p* < 0.05.

## 5 CONCLUSION

To our knowledge, this is the first study to research the general and standard method for obtaining high‐quality clinical‐grade hUC‐MSC subpopulations with specific markers. Five crucial factors need to be paid attention to and optimized simultaneously, constituting the five core steps of our standard operating procedures. Precise quality control at each step of the process of hUC‐MSC subpopulations can guarantee their functions while improving the efficiency of clinical transformation. Our optimized operation process can not only improve the enrichment efficiency of hUC‐MSCs subgroups and the reliability of preclinical research but also provide valuable and general methodological guidance for the rapid clinical transformation of specific MSC subpopulations.

## CONFLICT OF INTEREST

The authors indicate no potential conflicts of interest.

## Supporting information


**Figure S1** Characteristics and differentiation potential of clinical‐grade hUC‐MSCs.
(A) Representative optical, morphological images of primary clinical‐grade hUC‐MSCs of Passage 2 to Passage 5 derived from optimized tissue blocks (magnification: ×100). (B) Differentiation potential of hUC‐MSCs into mesodermal lineages. Representative images of hUC‐MSCs differentiated into adipocytes, osteocytes and chondrocytes are shown as indicated. Fat droplets were stained with Oil red O. Calcium phosphate deposits were stained with ALP and Alizarin Red. Proteoglycans with Toluidine Blue and Alcian Blue. (C) Flow cytometric analysis showed hUC‐MSCs were positive for mesenchymal lineage markers (CD73, CD90 and CD105), negative for haematopoietic and endothelial markers (CD34, CD45, CD19 and CD14), and negative for HLA‐DR. (D) Immunofluorescence staining of hUC‐MSCs showed they were positive for mesenchymal markers of α‐SMA (green) and Vimentin (red) and negative for epithelial markers of CK18 and E‐cadherin (scale bar = 100 μm).

**Figure S2** Taking the CD106+ subgroups as an example to optimize antibody incubation conditions and explore the optimal flow technology.
(A) Relationship between CD106 antibody volume and the proportion of MSC‐CD106^+^. (B) Relationship between CD106 antibody incubation time and the proportion of MSC‐CD106^+^. (C) The most advanced instrument and the suitable sorting conditions for the CD106 antibody were selected. (D) The flow sorting threshold gate was set to about 10% distance from the positive expression rate for the purity of the sorted CD106^+^ subpopulations.

**Figure S3** AO and PI were used to detect the viability of the cells.
AO and PI were used to detect the viability of the cells to label live cells and necrotic cells in the three groups, respectively. The lower right corner of Brightfield (BR) is the diameter distribution diagram (the abscissa is Cell Size/μm, and the ordinate is Count). The lower right corner of the AO and PI fluorescence is the fluorescence intensity distribution diagram (the abscissa is Relative Fluorescent Intensity/RFU, and the ordinate is Count).

**Figure S4** Schematic diagram of cell apoptosis and detection principle of dye combination.
Under normal circumstances, the phospholipid is specifically distributed in the inner lobes of the plasma membrane phospholipid bimolecular. The membrane potential of normal cells is stable. JC‐1 enters the mitochondria through the polarity of the mitochondrial membrane and forms a red fluorescent probe due to the increased concentration. Multimer, which is double‐positive for FL1 and FL2.In the early stage of apoptosis, PS flips from the inner lobes of the plasma membrane to the outer lobes of the plasma membrane. Annexin V is a Ca^+^ dependent phospholipid‐binding protein that can specifically bind to the PS that is flipped to the outer leaf of the plasma membrane with high affinity. FITC‐labelled Annexin V is used as a probe. At the same time, the mitochondrial transmembrane potential is depolarized, and JC‐1 is released from the mitochondria; the concentration is reduced, and it is reversed to a monomer form that emits green fluorescence, which is FL1 single positive. In the middle and late stages of apoptosis and dead cells, 7AAD can pass through the cell membrane and combine with the nucleus to appear red.

**Figure S5** After the three groups of cells were sorted, the growth changes of the cells from 0 to 84 h (Detailed in the supplement Video).
After flow sorting, CD106^+^ hUC‐MSCs from the three treatment groups were seeded and observed in real‐time using a living cell analyser and then took photos per 12 h. This video shows the photos of cells in three groups taken at different time points from 0 to 84 h (scale bar = 400 μm). The 7AAD and MACS groups gradually became denser compared with the CON group.

**Figure S6** Direct assessment for the vivo vascular‐angiogenic ability of the CD106^+^ hUC‐MSCs treated by the three methods after flow sorting.
(A) Macroscopic and microscopic view of matrigel plugs. The matrigel plug was harvested 21 days later. H & E staining was performed to reveal the vessel density in matrigel plug (scale bar = 100 μm or 50 μm). (B) Quantification of the vascular density of matrigel plugs was performed using Image J software (n = 3/group; all data shown as mean ± SEM).

**Table S1** Current reagents for removing apoptotic and necrotic cells.

## Data Availability

The data that support the findings of this study are available from the corresponding author upon reasonable request.
