# Supplementary material for: An optimized method for obtaining clinical‐grade specific cell subpopulations from human umbilical cord‐derived mesenchymal stem cells
Source: Cell Prolif. 2022 Jun 29;55(10):e13300. doi: 10.1111/cpr.13300 (PMC9528761; doi:10.1111/cpr.13300)
Supplement: Supplementary file 1 — Figure S1 Characteristics and differentiation potential of clinical‐grade hUC‐MSCs. (A) Representative optical, morphological images of primary clinical‐grade hUC‐MSCs of Passage 2 to Passage 5 derived from optimized tissue blocks (magnification: ×100). (B) Differentiation potential of hUC‐MSCs into mesodermal lineages. Representative images of hUC‐MSCs differentiated into adipocytes, osteocytes and chondrocytes are shown as indicated. Fat droplets were stained with Oil red O. Calcium phosphate deposits were stained with ALP and Alizarin Red. Proteoglycans with Toluidine Blue and Alcian Blue. (C) Flow cytometric analysis showed hUC‐MSCs were positive for mesenchymal lineage markers (CD73, CD90 and CD105), negative for haematopoietic and endothelial markers (CD34, CD45, CD19 and CD14), and negative for HLA‐DR. (D) Immunofluorescence staining of hUC‐MSCs showed they were positive for mesenchymal markers of α‐SMA (green) and Vimentin (red) and negative for epithelial markers of CK18 and E‐cadherin (scale bar = 100 μm). [file CPR-55-e13300-s007.zip › The raw data for Figure S1D and Figure S3 of Cellprol-6987-22.R1.pptx]

## Slide 1
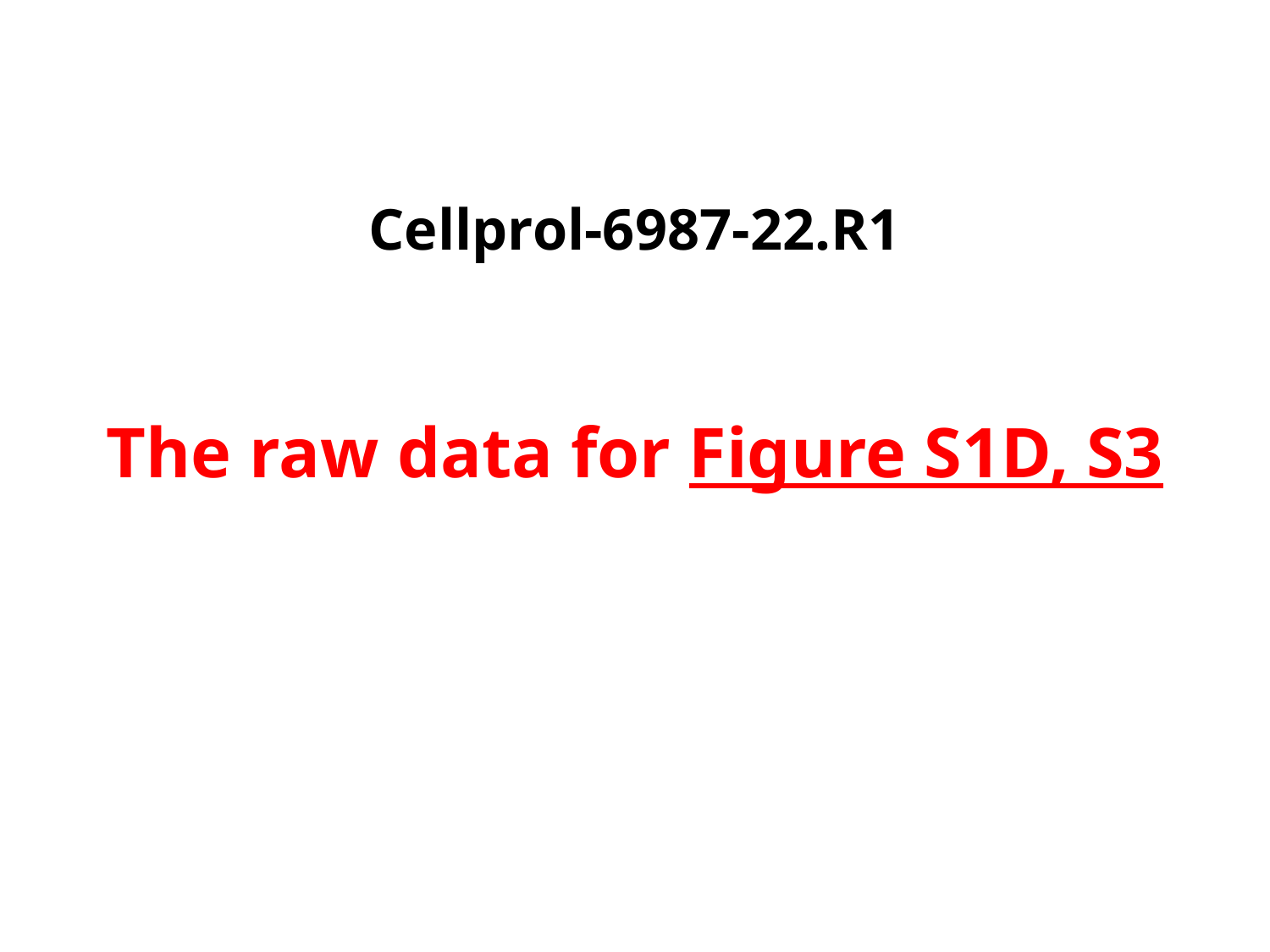

Cellprol-6987-22.R1
# The raw data for Figure S1D, S3

## Slide 2
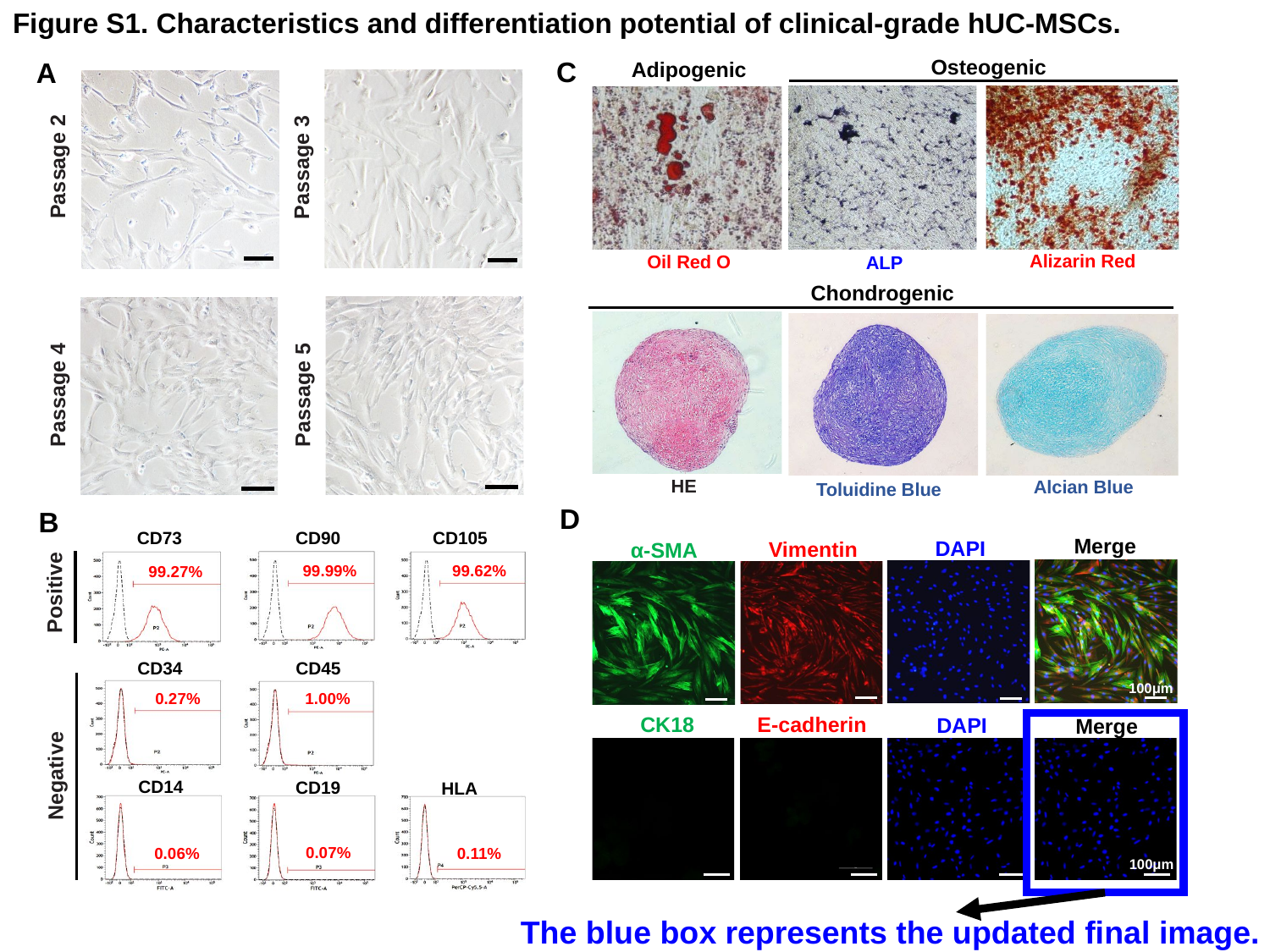

Figure S1. Characteristics and differentiation potential of clinical-grade hUC-MSCs.
Osteogenic
Adipogenic
Alizarin Red
Oil Red O
ALP
Chondrogenic
HE
Alcian Blue
Toluidine Blue
C
A
Passage 2
Passage 3
Passage 4
Passage 5
D
B
CD73
CD90
CD105
Positive
99.99%
99.62%
99.27%
CD34
CD45
Negative
0.27%
1.00%
CD14
CD19
HLA
0.07%
0.06%
0.11%
Merge
DAPI
Vimentin
α-SMA
100μm
CK18
E-cadherin
DAPI
Merge
100μm
100μm
The blue box represents the updated final image.

## Slide 3
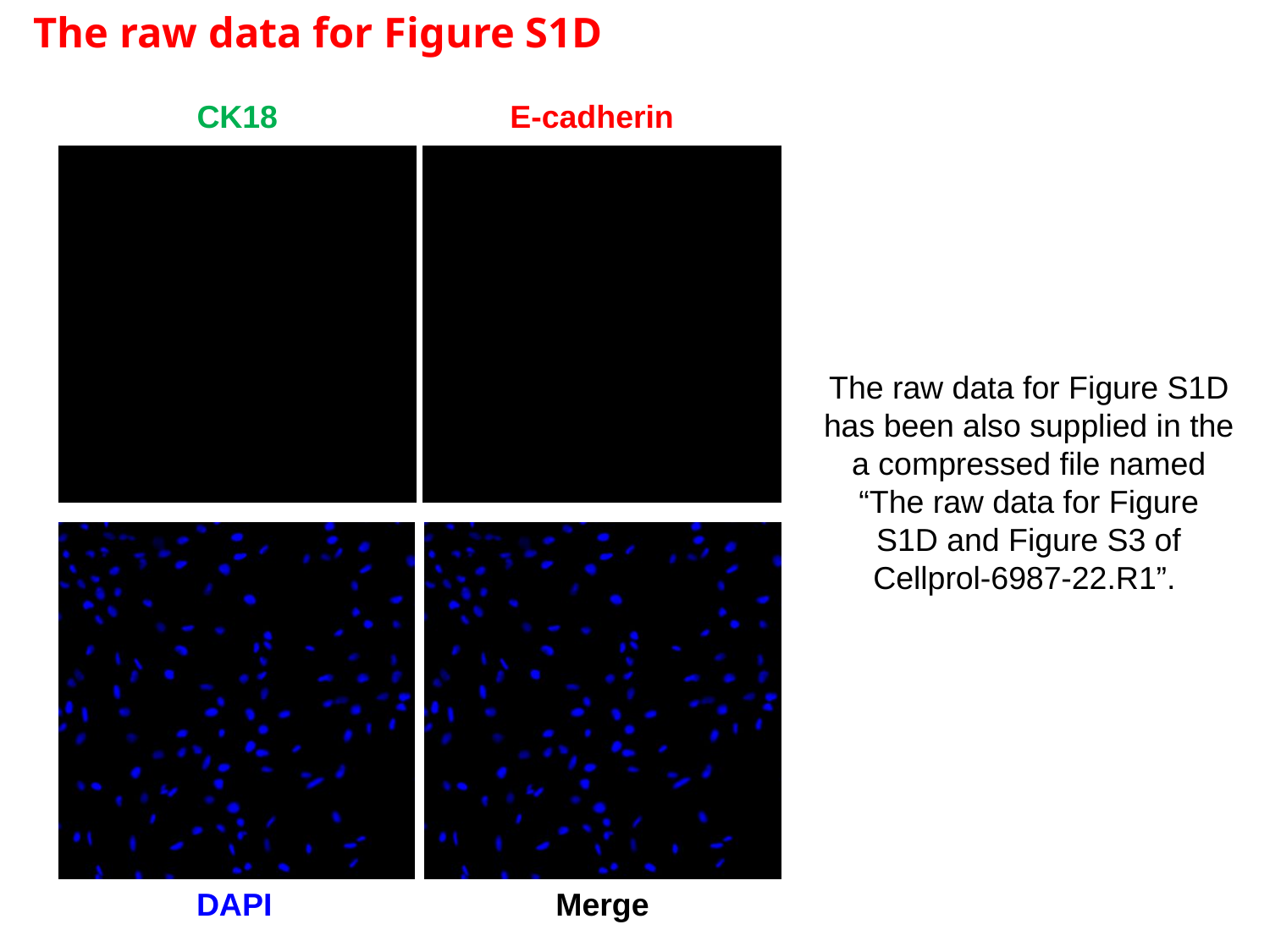

# The raw data for Figure S1D
CK18
E-cadherin
DAPI
Merge
The raw data for Figure S1D has been also supplied in the a compressed file named “The raw data for Figure S1D and Figure S3 of Cellprol-6987-22.R1”.

## Slide 4
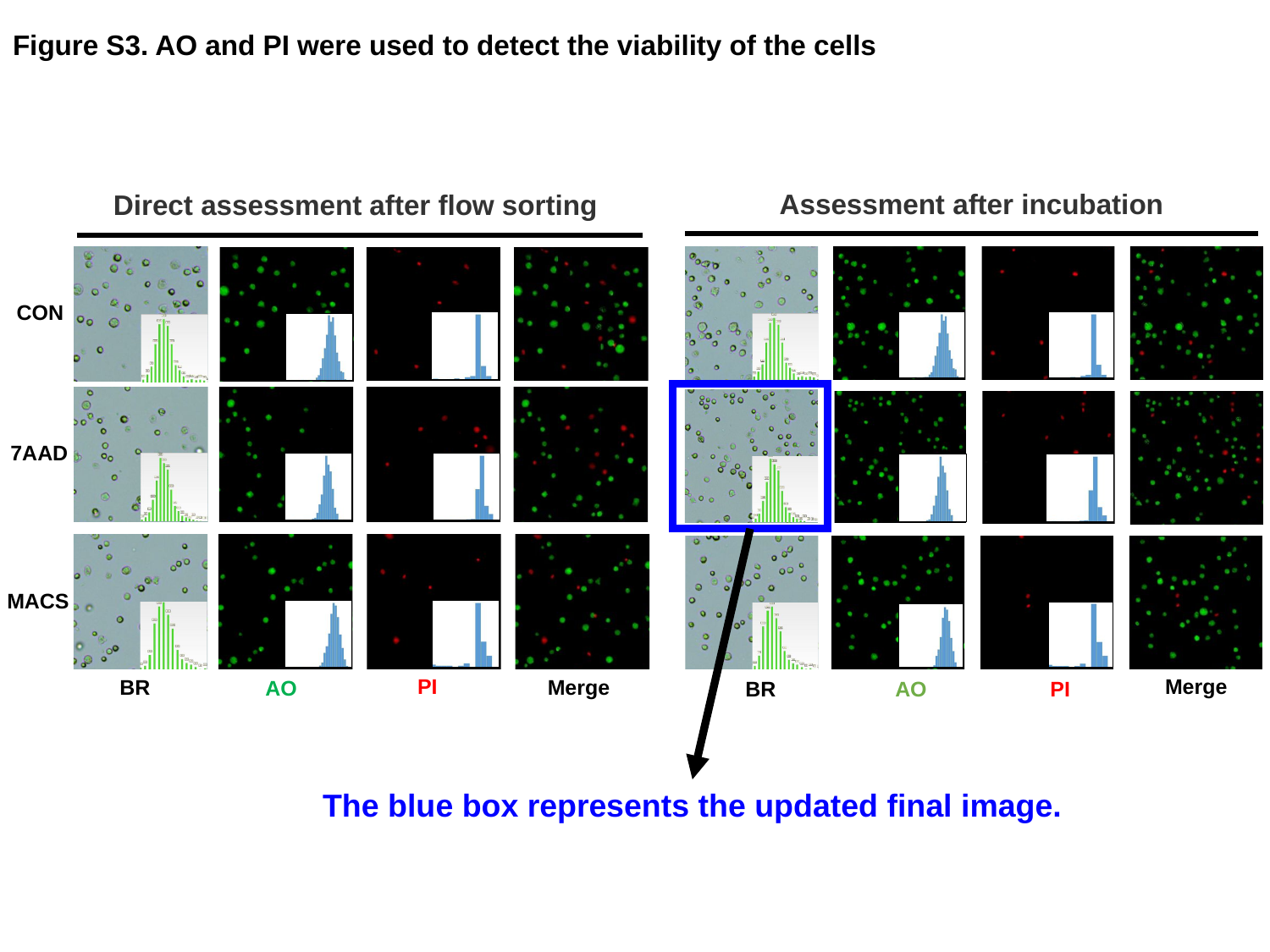

Figure S3. AO and PI were used to detect the viability of the cells
Assessment after incubation
Direct assessment after flow sorting
CON
7AAD
MACS
PI
Merge
Merge
BR
AO
PI
BR
AO
The blue box represents the updated final image.

## Slide 5
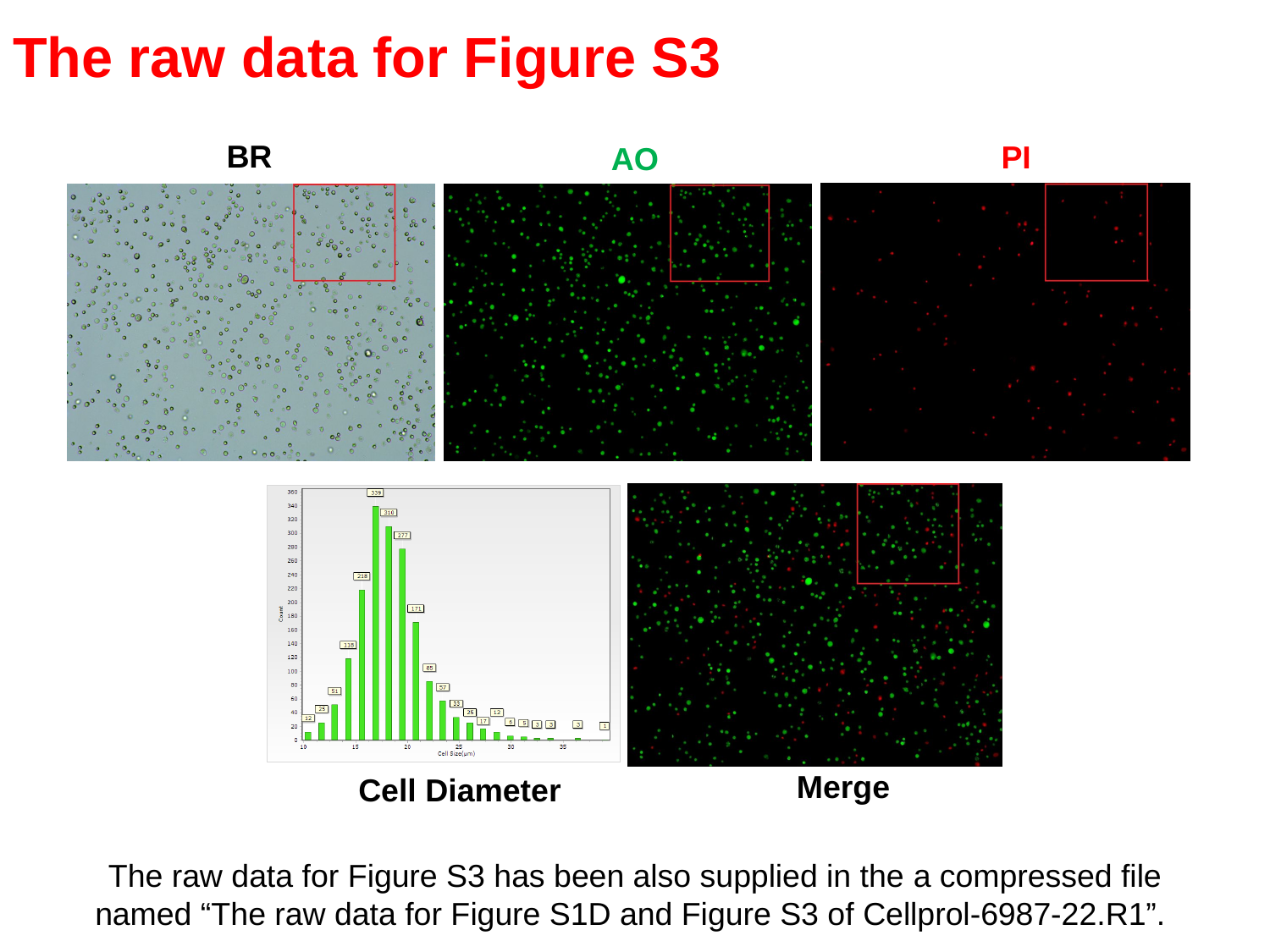

The raw data for Figure S3
BR
PI
AO
Merge
Cell Diameter
The raw data for Figure S3 has been also supplied in the a compressed file named “The raw data for Figure S1D and Figure S3 of Cellprol-6987-22.R1”.
