# Supplementary figures and images for: An optimized method for obtaining clinical‐grade specific cell subpopulations from human umbilical cord‐derived mesenchymal stem cells
Source: Cell Prolif. 2022 Jun 29;55(10):e13300. doi: 10.1111/cpr.13300 (PMC9528761; doi:10.1111/cpr.13300)

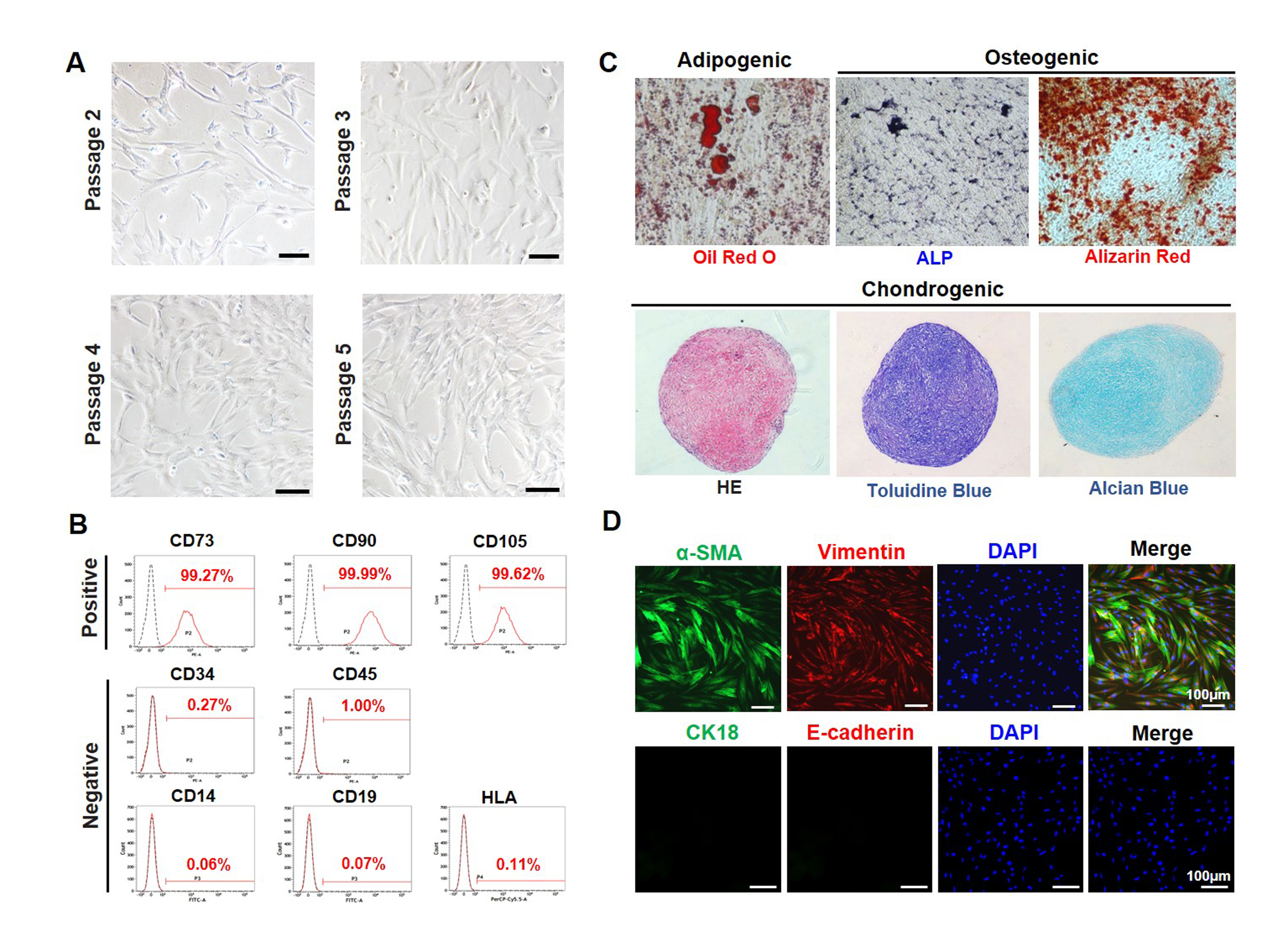

Supplement: Supplementary file 1 — Figure S1 Characteristics and differentiation potential of clinical‐grade hUC‐MSCs. (A) Representative optical, morphological images of primary clinical‐grade hUC‐MSCs of Passage 2 to Passage 5 derived from optimized tissue blocks (magnification: ×100). (B) Differentiation potential of hUC‐MSCs into mesodermal lineages. Representative images of hUC‐MSCs differentiated into adipocytes, osteocytes and chondrocytes are shown as indicated. Fat droplets were stained with Oil red O. Calcium phosphate deposits were stained with ALP and Alizarin Red. Proteoglycans with Toluidine Blue and Alcian Blue. (C) Flow cytometric analysis showed hUC‐MSCs were positive for mesenchymal lineage markers (CD73, CD90 and CD105), negative for haematopoietic and endothelial markers (CD34, CD45, CD19 and CD14), and negative for HLA‐DR. (D) Immunofluorescence staining of hUC‐MSCs showed they were positive for mesenchymal markers of α‐SMA (green) and Vimentin (red) and negative for epithelial markers of CK18 and E‐cadherin (scale bar = 100 μm). [file CPR-55-e13300-s007.zip › Figure S1.tif]

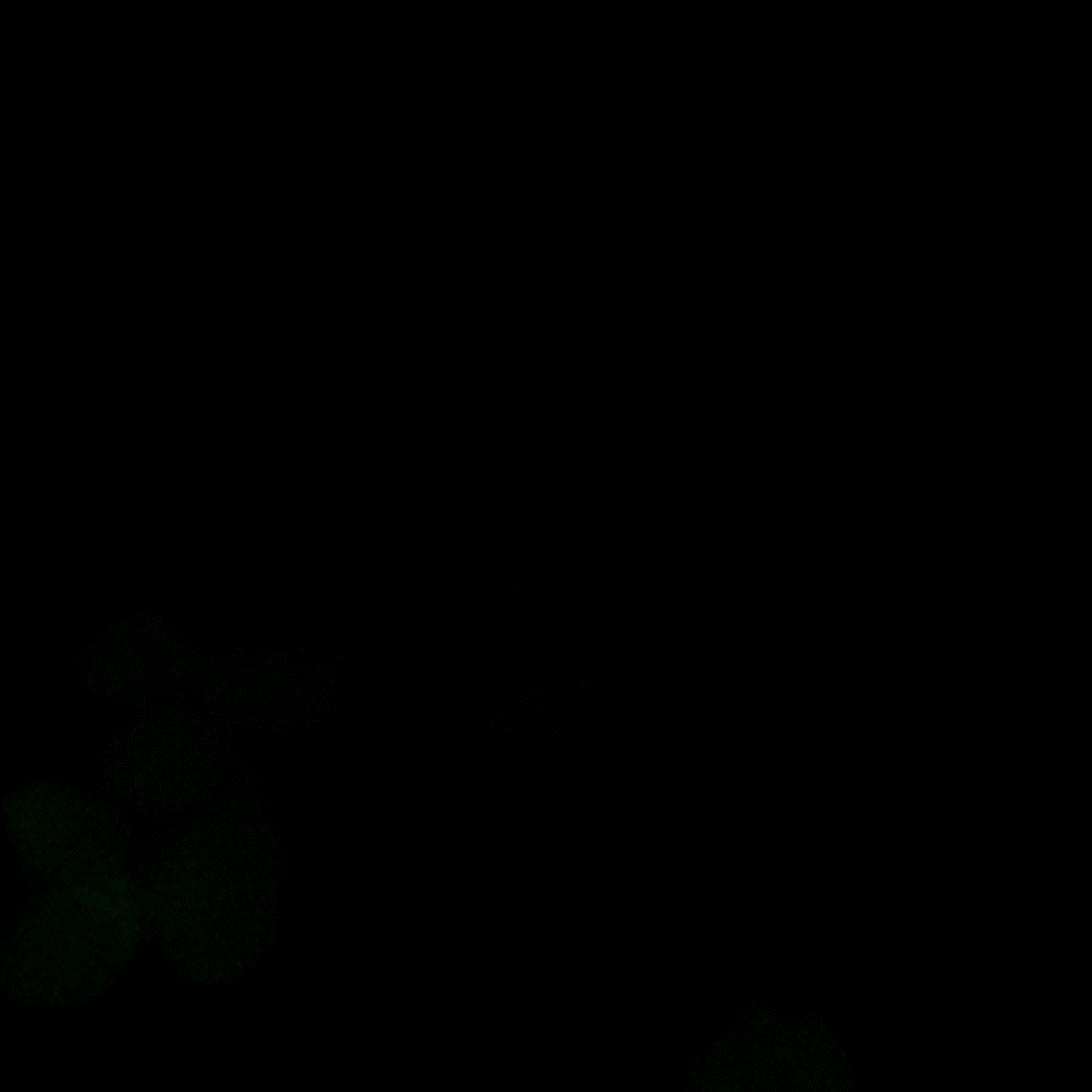

Supplement: Supplementary file 1 — Figure S1 Characteristics and differentiation potential of clinical‐grade hUC‐MSCs. (A) Representative optical, morphological images of primary clinical‐grade hUC‐MSCs of Passage 2 to Passage 5 derived from optimized tissue blocks (magnification: ×100). (B) Differentiation potential of hUC‐MSCs into mesodermal lineages. Representative images of hUC‐MSCs differentiated into adipocytes, osteocytes and chondrocytes are shown as indicated. Fat droplets were stained with Oil red O. Calcium phosphate deposits were stained with ALP and Alizarin Red. Proteoglycans with Toluidine Blue and Alcian Blue. (C) Flow cytometric analysis showed hUC‐MSCs were positive for mesenchymal lineage markers (CD73, CD90 and CD105), negative for haematopoietic and endothelial markers (CD34, CD45, CD19 and CD14), and negative for HLA‐DR. (D) Immunofluorescence staining of hUC‐MSCs showed they were positive for mesenchymal markers of α‐SMA (green) and Vimentin (red) and negative for epithelial markers of CK18 and E‐cadherin (scale bar = 100 μm). [file CPR-55-e13300-s007.zip › Figure S1D--CK18.png]

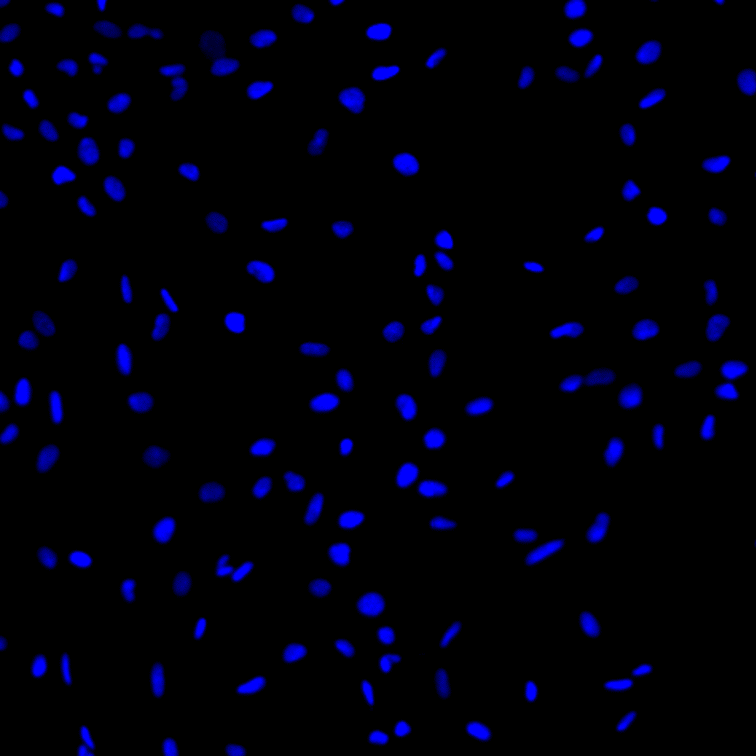

Supplement: Supplementary file 1 — Figure S1 Characteristics and differentiation potential of clinical‐grade hUC‐MSCs. (A) Representative optical, morphological images of primary clinical‐grade hUC‐MSCs of Passage 2 to Passage 5 derived from optimized tissue blocks (magnification: ×100). (B) Differentiation potential of hUC‐MSCs into mesodermal lineages. Representative images of hUC‐MSCs differentiated into adipocytes, osteocytes and chondrocytes are shown as indicated. Fat droplets were stained with Oil red O. Calcium phosphate deposits were stained with ALP and Alizarin Red. Proteoglycans with Toluidine Blue and Alcian Blue. (C) Flow cytometric analysis showed hUC‐MSCs were positive for mesenchymal lineage markers (CD73, CD90 and CD105), negative for haematopoietic and endothelial markers (CD34, CD45, CD19 and CD14), and negative for HLA‐DR. (D) Immunofluorescence staining of hUC‐MSCs showed they were positive for mesenchymal markers of α‐SMA (green) and Vimentin (red) and negative for epithelial markers of CK18 and E‐cadherin (scale bar = 100 μm). [file CPR-55-e13300-s007.zip › Figure S1D--DAPI.png]

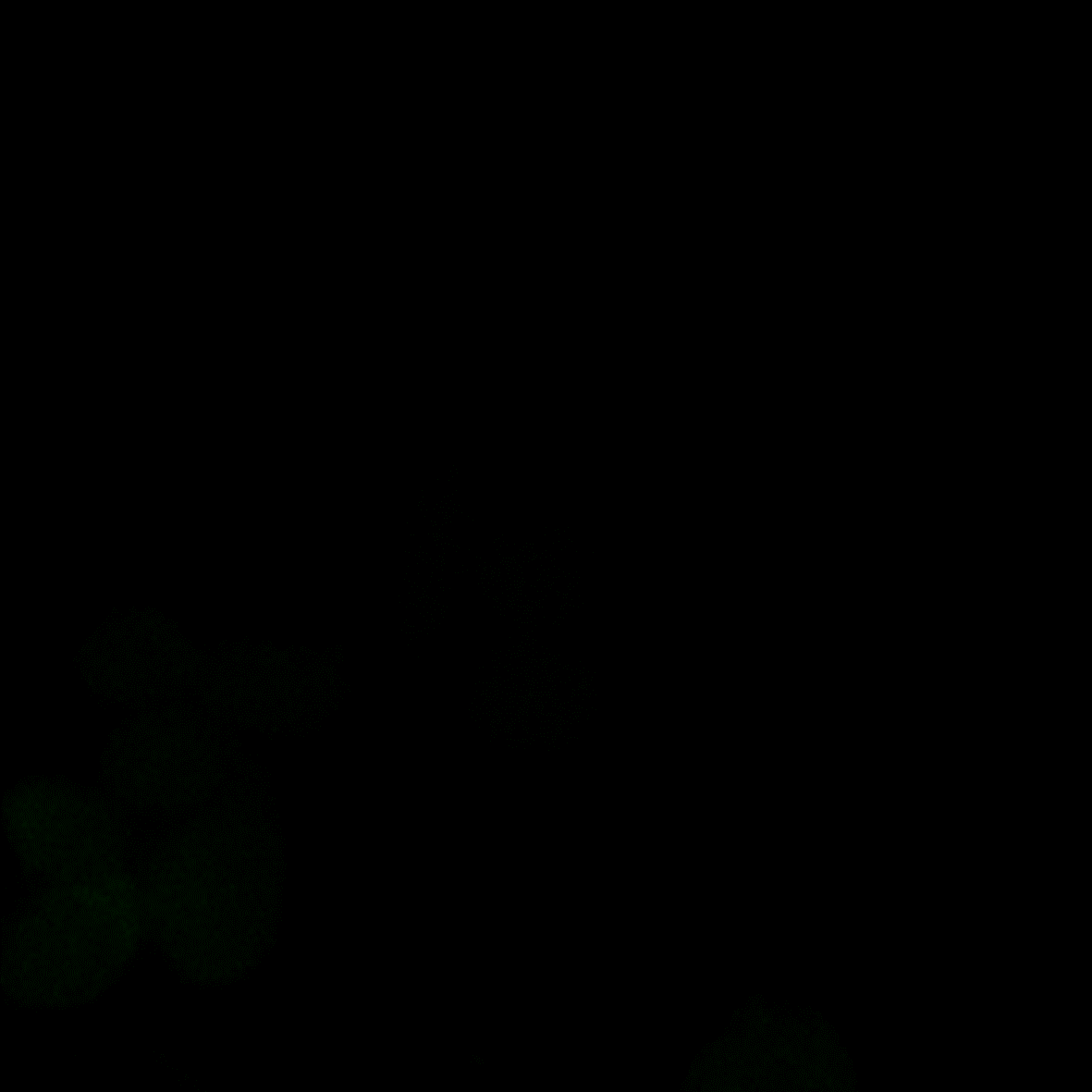

Supplement: Supplementary file 1 — Figure S1 Characteristics and differentiation potential of clinical‐grade hUC‐MSCs. (A) Representative optical, morphological images of primary clinical‐grade hUC‐MSCs of Passage 2 to Passage 5 derived from optimized tissue blocks (magnification: ×100). (B) Differentiation potential of hUC‐MSCs into mesodermal lineages. Representative images of hUC‐MSCs differentiated into adipocytes, osteocytes and chondrocytes are shown as indicated. Fat droplets were stained with Oil red O. Calcium phosphate deposits were stained with ALP and Alizarin Red. Proteoglycans with Toluidine Blue and Alcian Blue. (C) Flow cytometric analysis showed hUC‐MSCs were positive for mesenchymal lineage markers (CD73, CD90 and CD105), negative for haematopoietic and endothelial markers (CD34, CD45, CD19 and CD14), and negative for HLA‐DR. (D) Immunofluorescence staining of hUC‐MSCs showed they were positive for mesenchymal markers of α‐SMA (green) and Vimentin (red) and negative for epithelial markers of CK18 and E‐cadherin (scale bar = 100 μm). [file CPR-55-e13300-s007.zip › Figure S1D--E-cadherin.png]

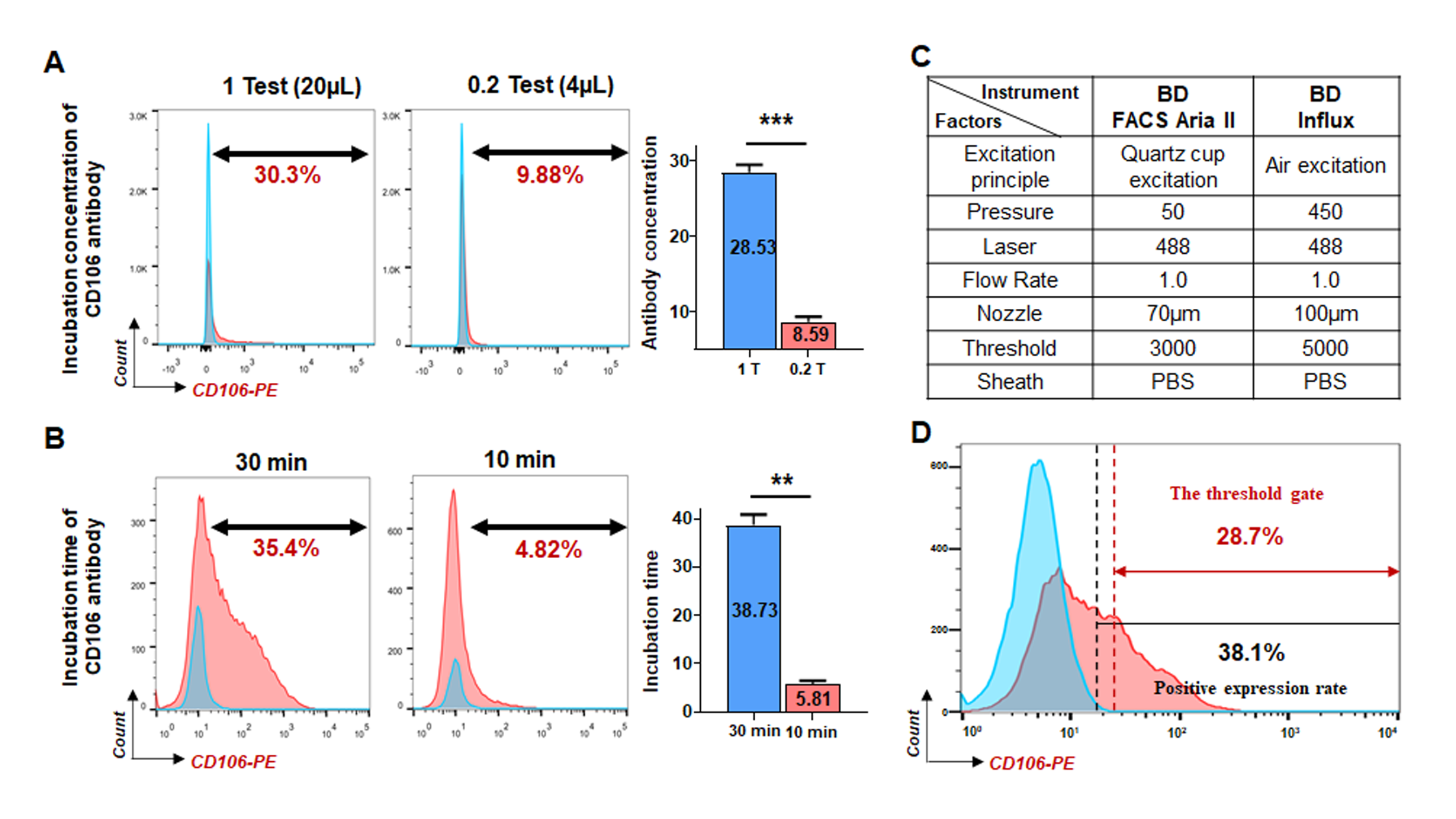

Supplement: Supplementary file 2 — Figure S2 Taking the CD106+ subgroups as an example to optimize antibody incubation conditions and explore the optimal flow technology. (A) Relationship between CD106 antibody volume and the proportion of MSC‐CD106+. (B) Relationship between CD106 antibody incubation time and the proportion of MSC‐CD106+. (C) The most advanced instrument and the suitable sorting conditions for the CD106 antibody were selected. (D) The flow sorting threshold gate was set to about 10% distance from the positive expression rate for the purity of the sorted CD106+ subpopulations. [file CPR-55-e13300-s003.tif]

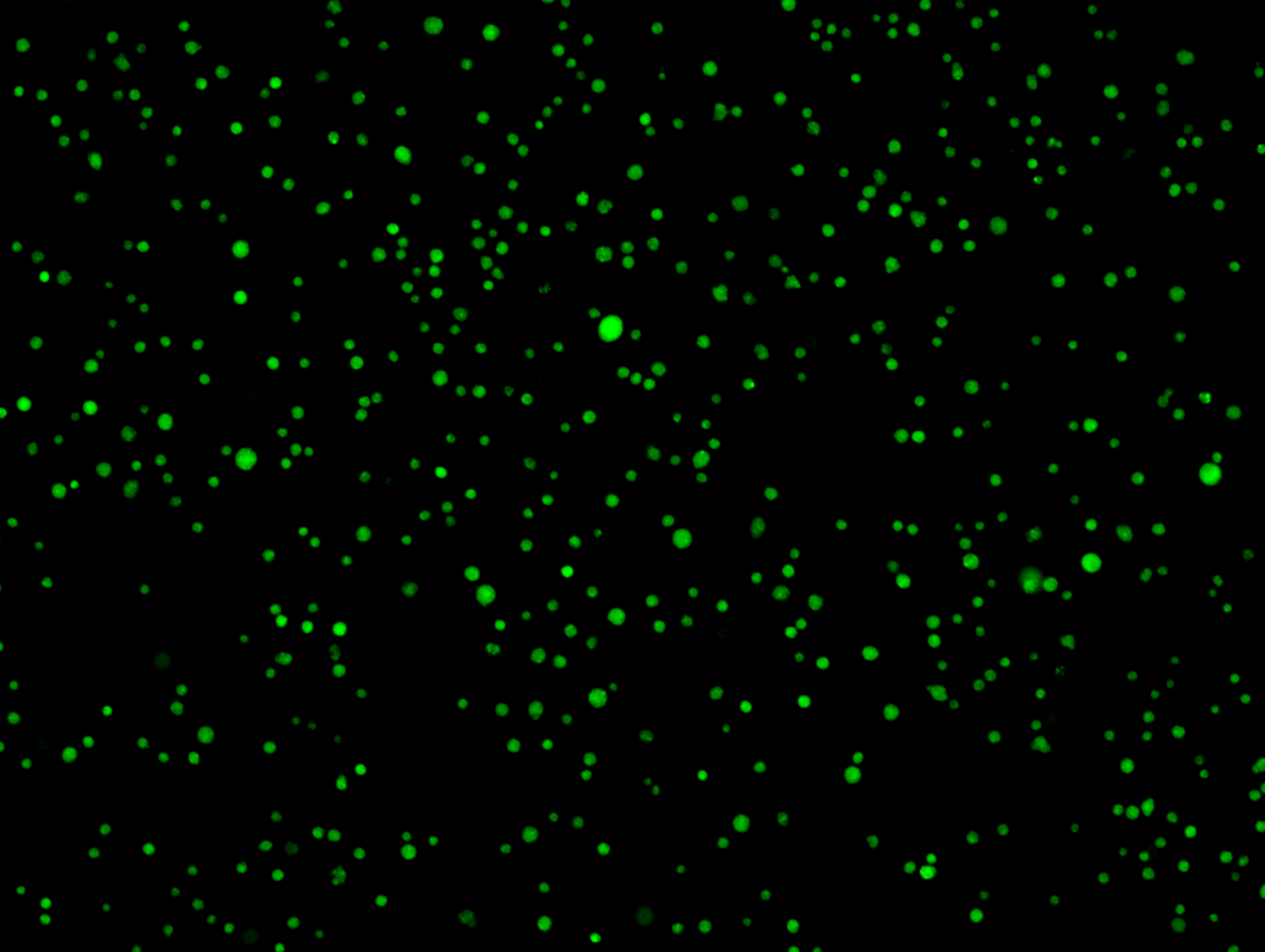

Supplement: Supplementary file 3 — Figure S3 AO and PI were used to detect the viability of the cells. AO and PI were used to detect the viability of the cells to label live cells and necrotic cells in the three groups, respectively. The lower right corner of Brightfield (BR) is the diameter distribution diagram (the abscissa is Cell Size/μm, and the ordinate is Count). The lower right corner of the AO and PI fluorescence is the fluorescence intensity distribution diagram (the abscissa is Relative Fluorescent Intensity/RFU, and the ordinate is Count). [file CPR-55-e13300-s001.zip › AO (Figure S3-Assessment after incubation-7AAD).png]

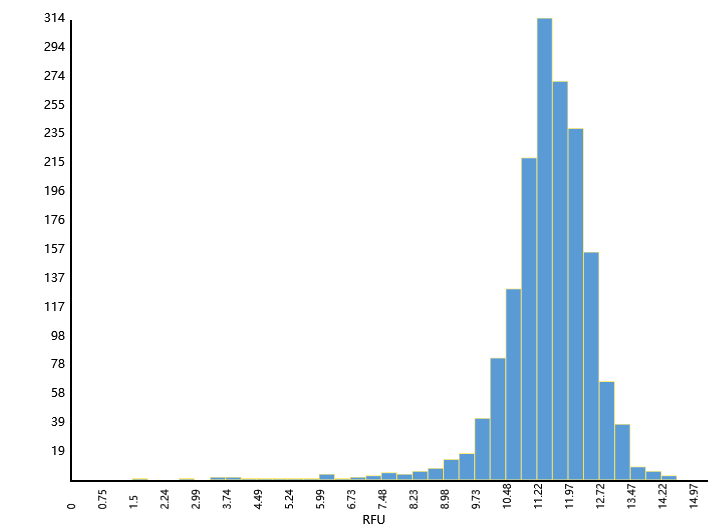

Supplement: Supplementary file 3 — Figure S3 AO and PI were used to detect the viability of the cells. AO and PI were used to detect the viability of the cells to label live cells and necrotic cells in the three groups, respectively. The lower right corner of Brightfield (BR) is the diameter distribution diagram (the abscissa is Cell Size/μm, and the ordinate is Count). The lower right corner of the AO and PI fluorescence is the fluorescence intensity distribution diagram (the abscissa is Relative Fluorescent Intensity/RFU, and the ordinate is Count). [file CPR-55-e13300-s001.zip › AO fluorescence intensity (Figure S3-Assessment after incubation-7AAD).png]

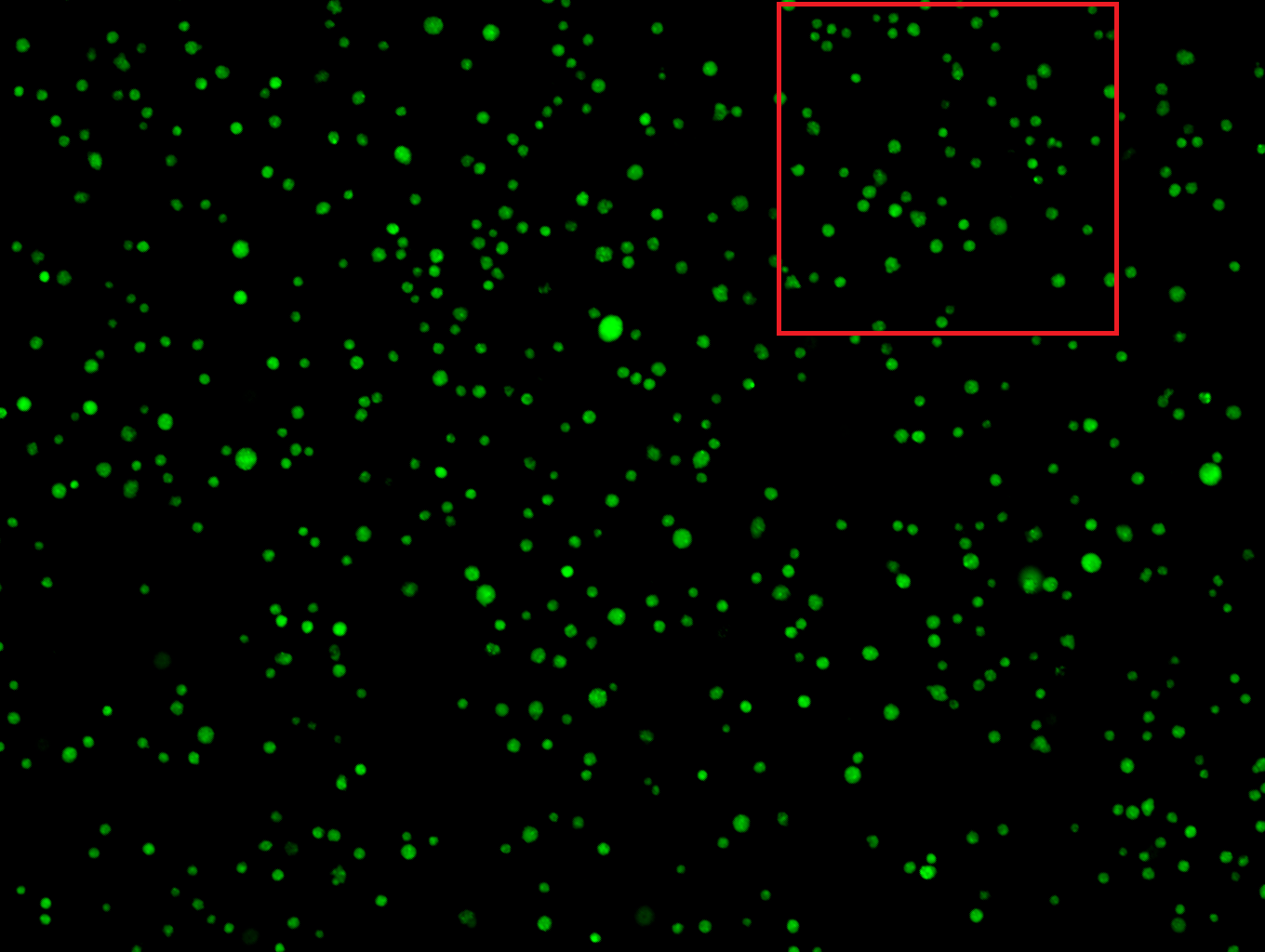

Supplement: Supplementary file 3 — Figure S3 AO and PI were used to detect the viability of the cells. AO and PI were used to detect the viability of the cells to label live cells and necrotic cells in the three groups, respectively. The lower right corner of Brightfield (BR) is the diameter distribution diagram (the abscissa is Cell Size/μm, and the ordinate is Count). The lower right corner of the AO and PI fluorescence is the fluorescence intensity distribution diagram (the abscissa is Relative Fluorescent Intensity/RFU, and the ordinate is Count). [file CPR-55-e13300-s001.zip › AO-selected view (Figure S3-Assessment after incubation-7AAD).png]

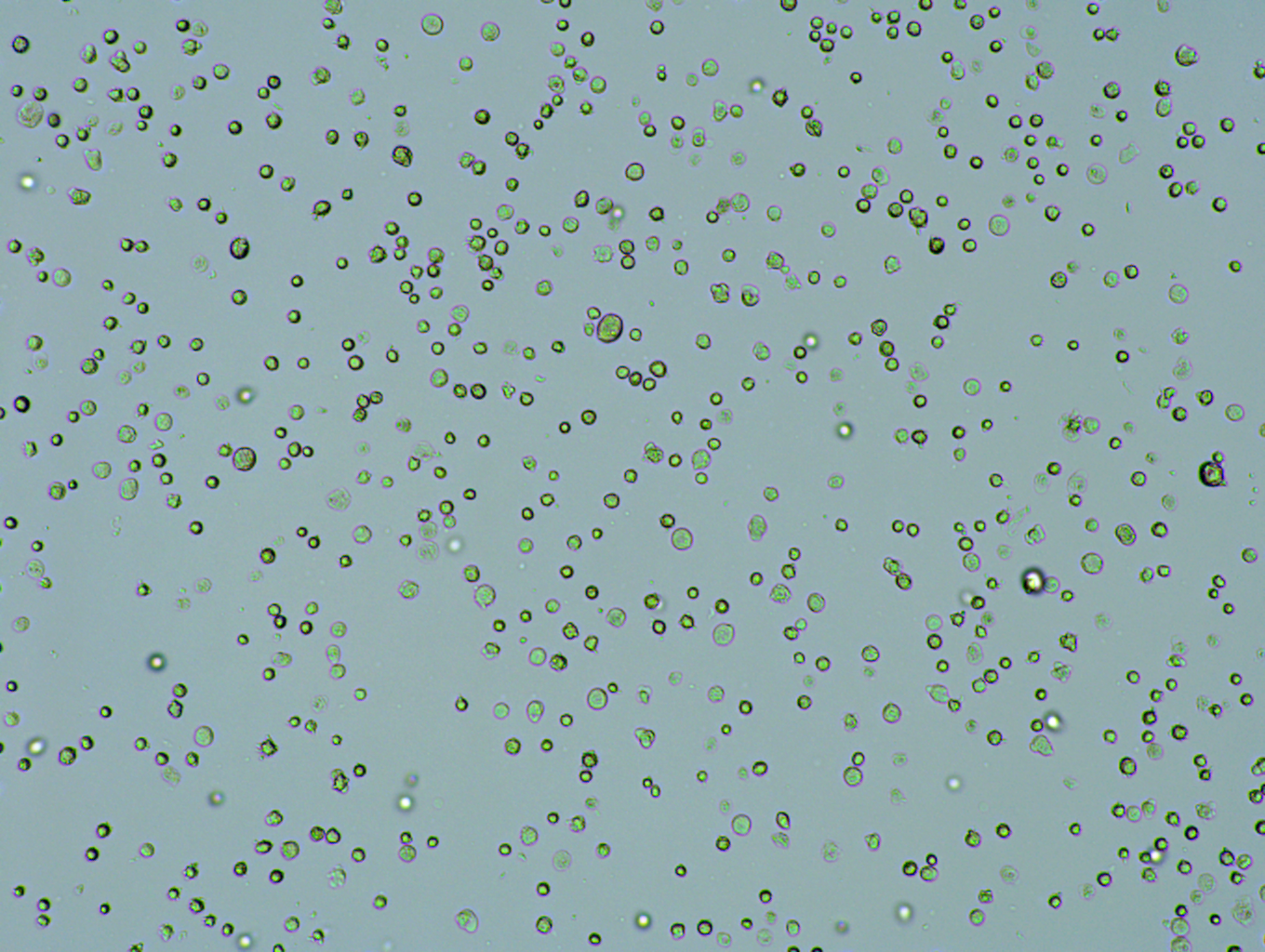

Supplement: Supplementary file 3 — Figure S3 AO and PI were used to detect the viability of the cells. AO and PI were used to detect the viability of the cells to label live cells and necrotic cells in the three groups, respectively. The lower right corner of Brightfield (BR) is the diameter distribution diagram (the abscissa is Cell Size/μm, and the ordinate is Count). The lower right corner of the AO and PI fluorescence is the fluorescence intensity distribution diagram (the abscissa is Relative Fluorescent Intensity/RFU, and the ordinate is Count). [file CPR-55-e13300-s001.zip › BR (Figure S3-Assessment after incubation-7AAD).jpg]

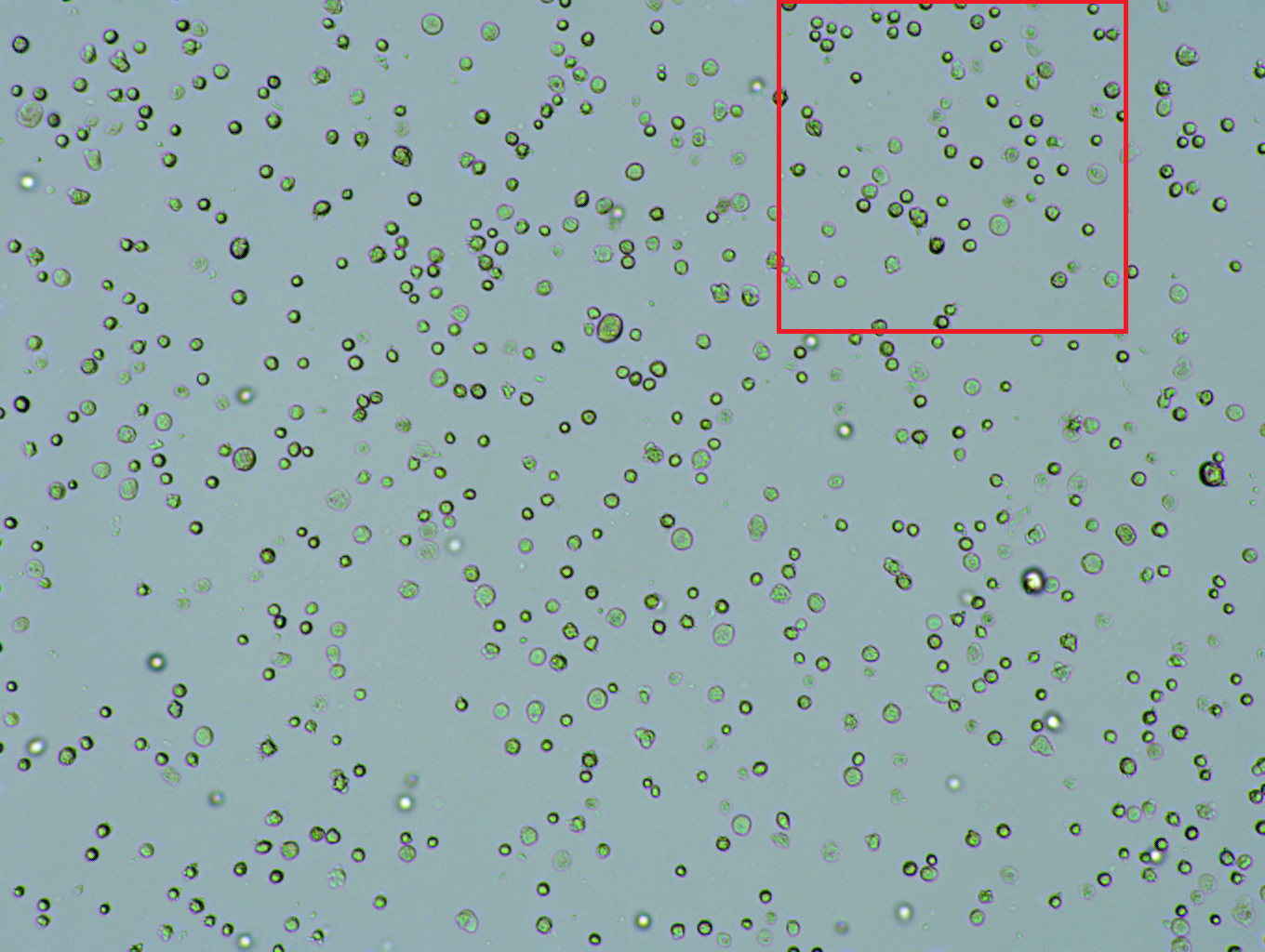

Supplement: Supplementary file 3 — Figure S3 AO and PI were used to detect the viability of the cells. AO and PI were used to detect the viability of the cells to label live cells and necrotic cells in the three groups, respectively. The lower right corner of Brightfield (BR) is the diameter distribution diagram (the abscissa is Cell Size/μm, and the ordinate is Count). The lower right corner of the AO and PI fluorescence is the fluorescence intensity distribution diagram (the abscissa is Relative Fluorescent Intensity/RFU, and the ordinate is Count). [file CPR-55-e13300-s001.zip › BR-selected view (Figure S3-Assessment after incubation-7AAD).jpg]

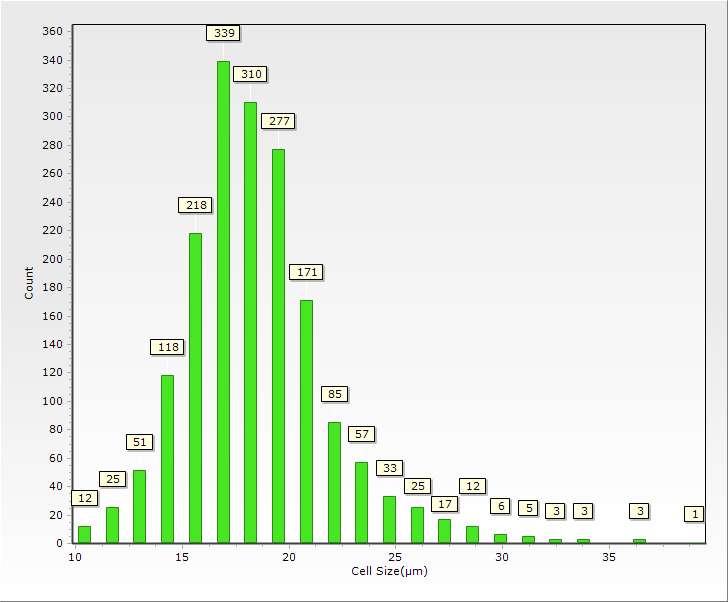

Supplement: Supplementary file 3 — Figure S3 AO and PI were used to detect the viability of the cells. AO and PI were used to detect the viability of the cells to label live cells and necrotic cells in the three groups, respectively. The lower right corner of Brightfield (BR) is the diameter distribution diagram (the abscissa is Cell Size/μm, and the ordinate is Count). The lower right corner of the AO and PI fluorescence is the fluorescence intensity distribution diagram (the abscissa is Relative Fluorescent Intensity/RFU, and the ordinate is Count). [file CPR-55-e13300-s001.zip › Cell Diameter (Figure S3-Assessment after incubation-7AAD).png]

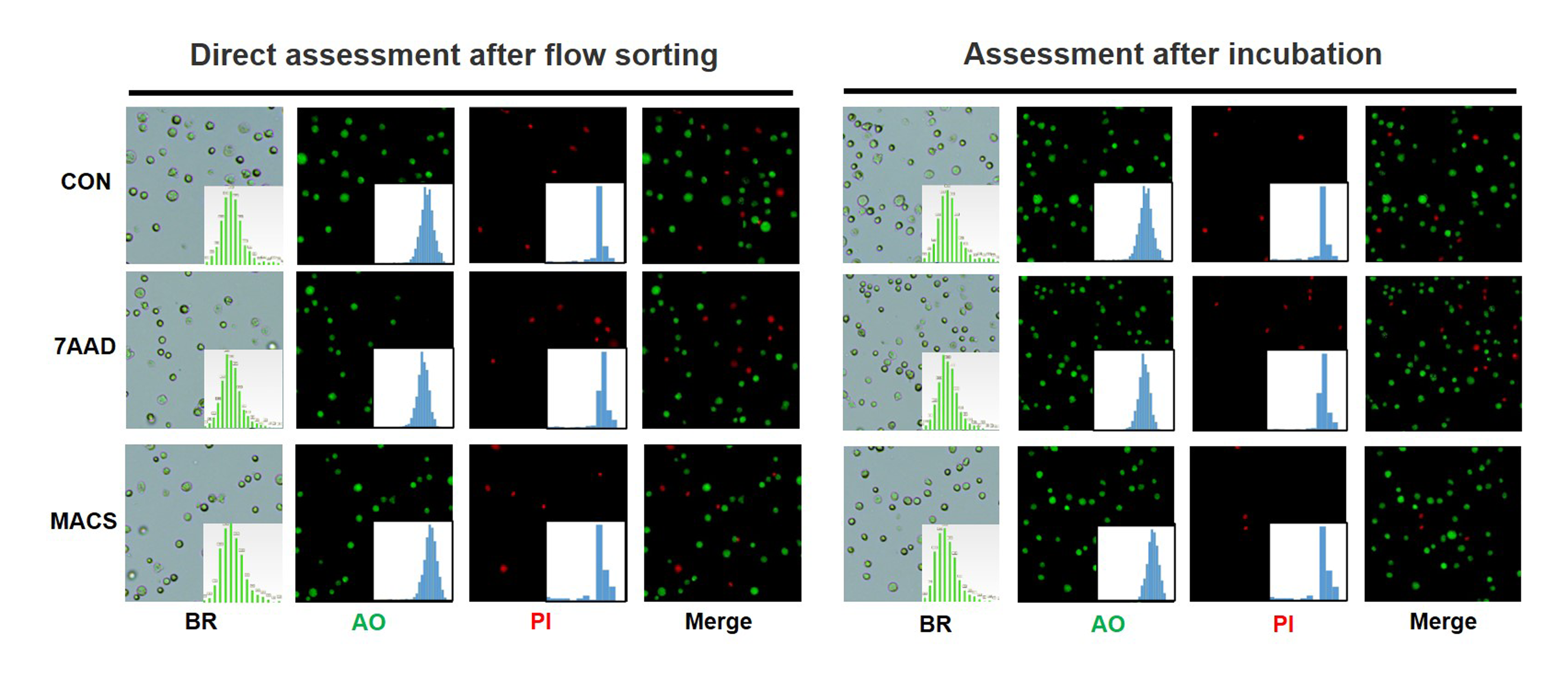

Supplement: Supplementary file 3 — Figure S3 AO and PI were used to detect the viability of the cells. AO and PI were used to detect the viability of the cells to label live cells and necrotic cells in the three groups, respectively. The lower right corner of Brightfield (BR) is the diameter distribution diagram (the abscissa is Cell Size/μm, and the ordinate is Count). The lower right corner of the AO and PI fluorescence is the fluorescence intensity distribution diagram (the abscissa is Relative Fluorescent Intensity/RFU, and the ordinate is Count). [file CPR-55-e13300-s001.zip › Figure S3.tif]

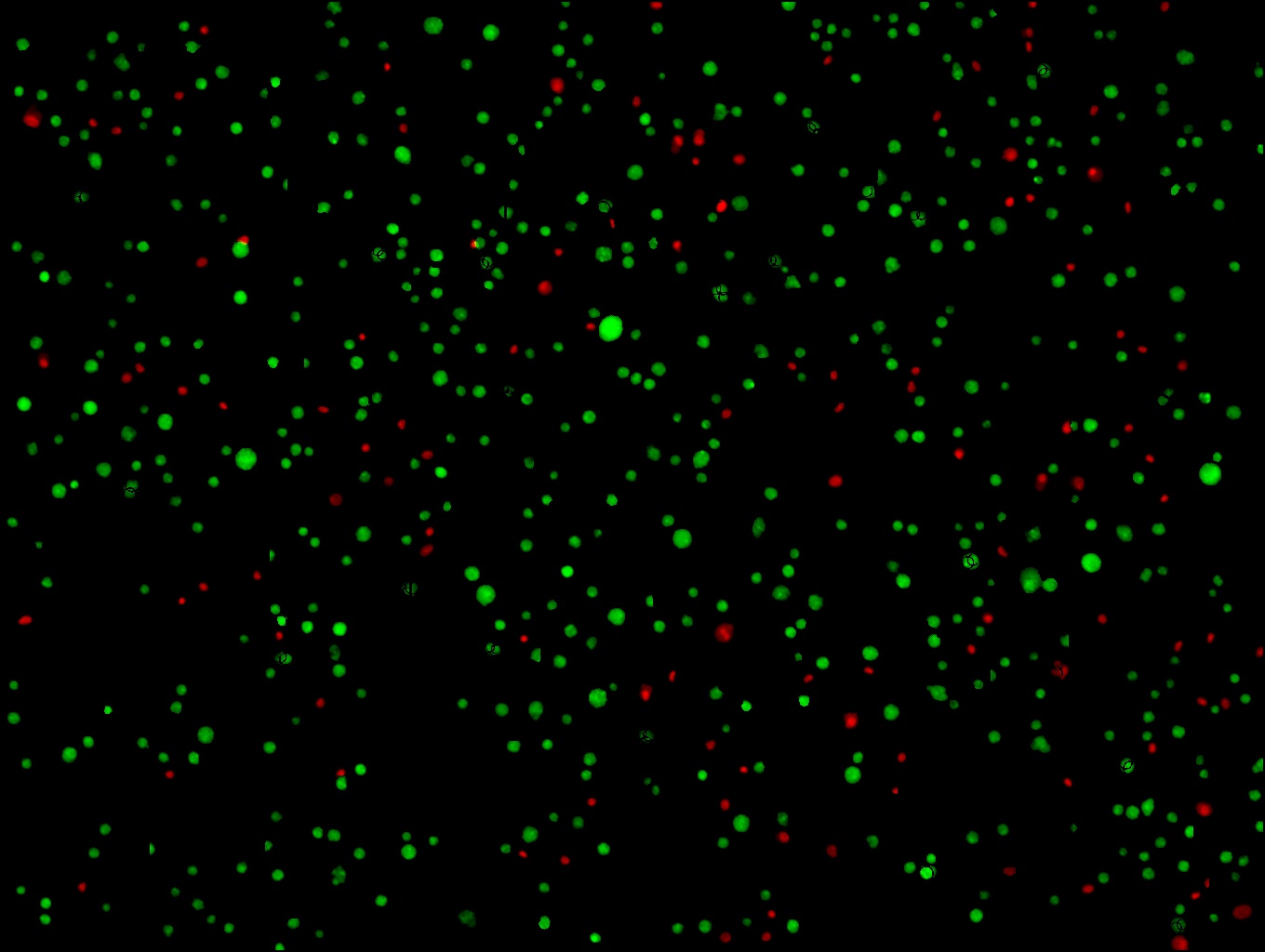

Supplement: Supplementary file 3 — Figure S3 AO and PI were used to detect the viability of the cells. AO and PI were used to detect the viability of the cells to label live cells and necrotic cells in the three groups, respectively. The lower right corner of Brightfield (BR) is the diameter distribution diagram (the abscissa is Cell Size/μm, and the ordinate is Count). The lower right corner of the AO and PI fluorescence is the fluorescence intensity distribution diagram (the abscissa is Relative Fluorescent Intensity/RFU, and the ordinate is Count). [file CPR-55-e13300-s001.zip › Merge (Figure S3-Assessment after incubation-7AAD).jpg]

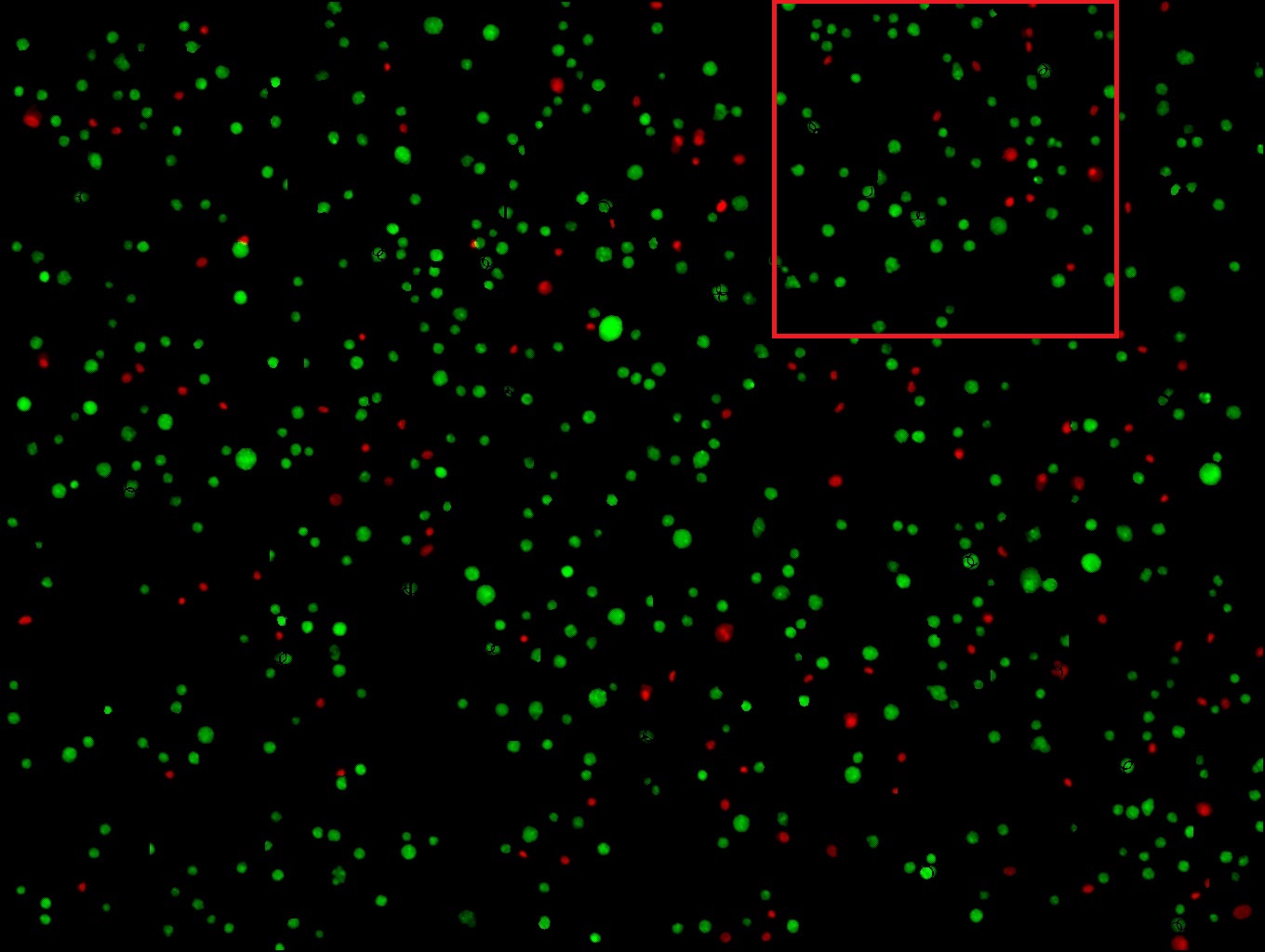

Supplement: Supplementary file 3 — Figure S3 AO and PI were used to detect the viability of the cells. AO and PI were used to detect the viability of the cells to label live cells and necrotic cells in the three groups, respectively. The lower right corner of Brightfield (BR) is the diameter distribution diagram (the abscissa is Cell Size/μm, and the ordinate is Count). The lower right corner of the AO and PI fluorescence is the fluorescence intensity distribution diagram (the abscissa is Relative Fluorescent Intensity/RFU, and the ordinate is Count). [file CPR-55-e13300-s001.zip › Merge-selected view (Figure S3-Assessment after incubation-7AAD).jpg]

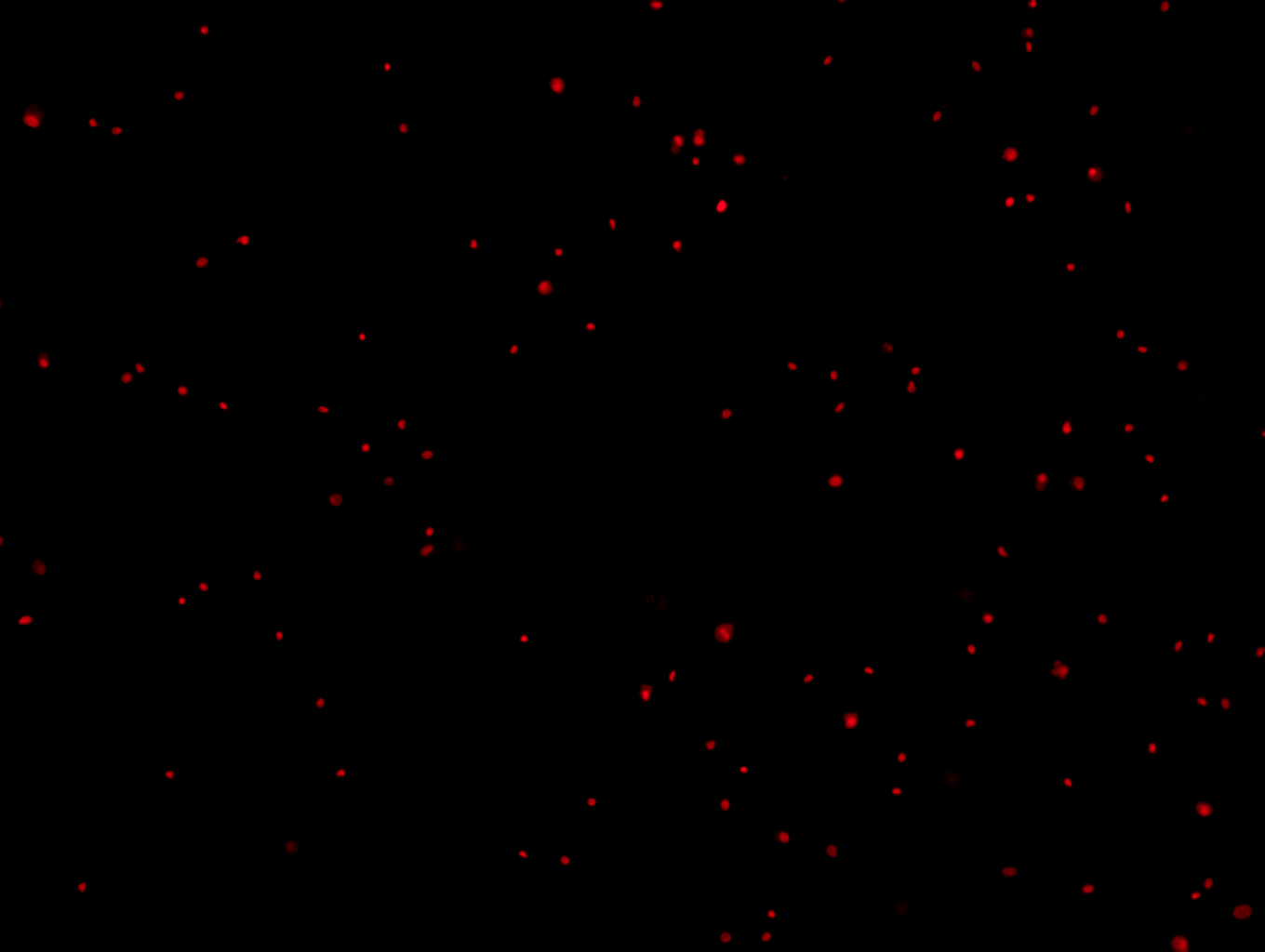

Supplement: Supplementary file 3 — Figure S3 AO and PI were used to detect the viability of the cells. AO and PI were used to detect the viability of the cells to label live cells and necrotic cells in the three groups, respectively. The lower right corner of Brightfield (BR) is the diameter distribution diagram (the abscissa is Cell Size/μm, and the ordinate is Count). The lower right corner of the AO and PI fluorescence is the fluorescence intensity distribution diagram (the abscissa is Relative Fluorescent Intensity/RFU, and the ordinate is Count). [file CPR-55-e13300-s001.zip › PI (Figure S3-Assessment after incubation-7AAD).png]

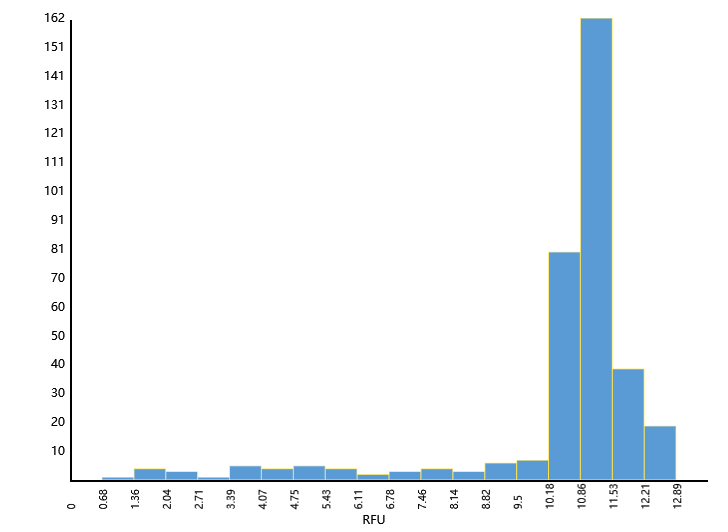

Supplement: Supplementary file 3 — Figure S3 AO and PI were used to detect the viability of the cells. AO and PI were used to detect the viability of the cells to label live cells and necrotic cells in the three groups, respectively. The lower right corner of Brightfield (BR) is the diameter distribution diagram (the abscissa is Cell Size/μm, and the ordinate is Count). The lower right corner of the AO and PI fluorescence is the fluorescence intensity distribution diagram (the abscissa is Relative Fluorescent Intensity/RFU, and the ordinate is Count). [file CPR-55-e13300-s001.zip › PI fluorescence intensity (Figure S3-Assessment after incubation-7AAD).png]

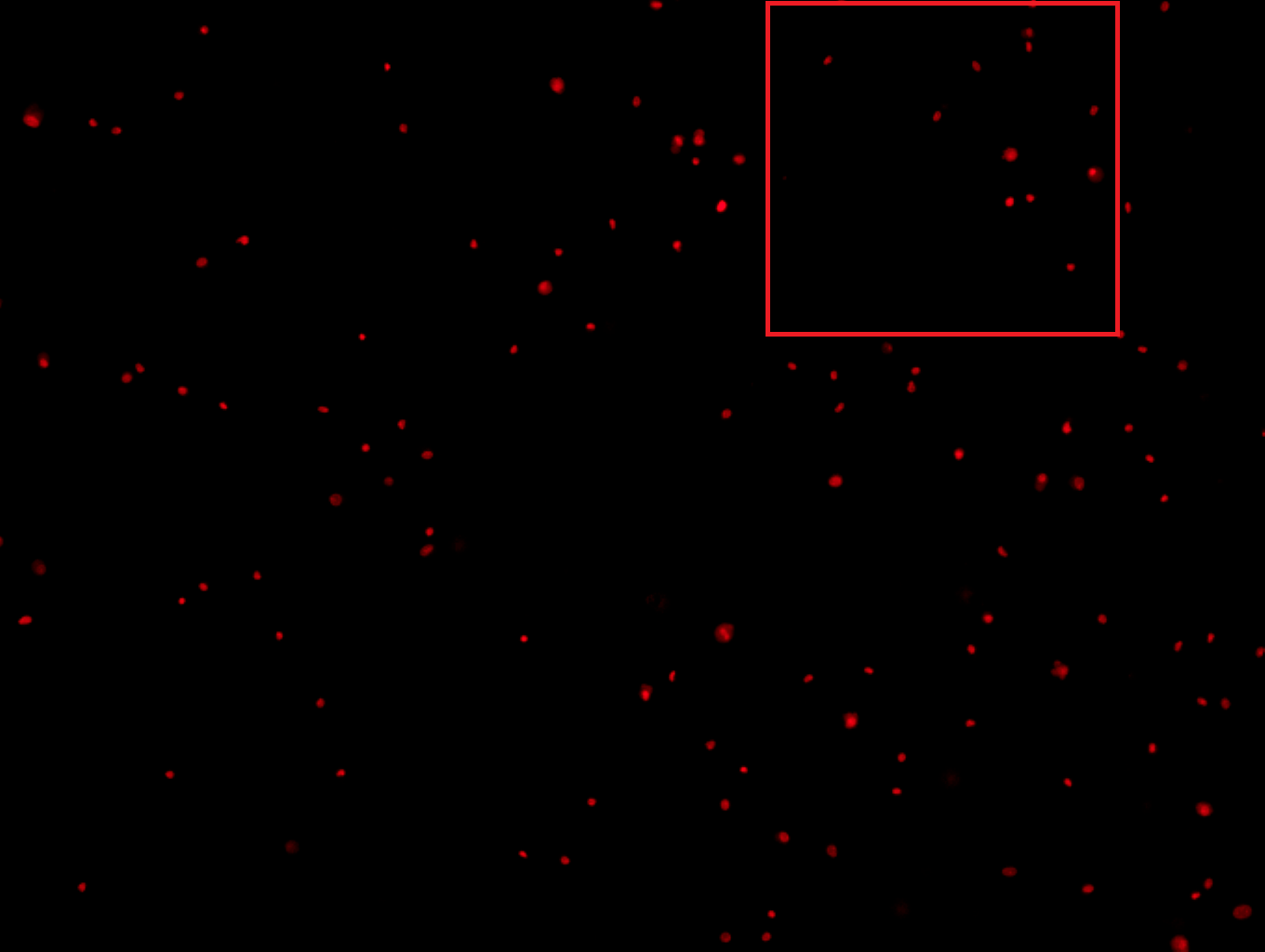

Supplement: Supplementary file 3 — Figure S3 AO and PI were used to detect the viability of the cells. AO and PI were used to detect the viability of the cells to label live cells and necrotic cells in the three groups, respectively. The lower right corner of Brightfield (BR) is the diameter distribution diagram (the abscissa is Cell Size/μm, and the ordinate is Count). The lower right corner of the AO and PI fluorescence is the fluorescence intensity distribution diagram (the abscissa is Relative Fluorescent Intensity/RFU, and the ordinate is Count). [file CPR-55-e13300-s001.zip › PI--selected view (Figure S3-Assessment after incubation-7AAD).png]

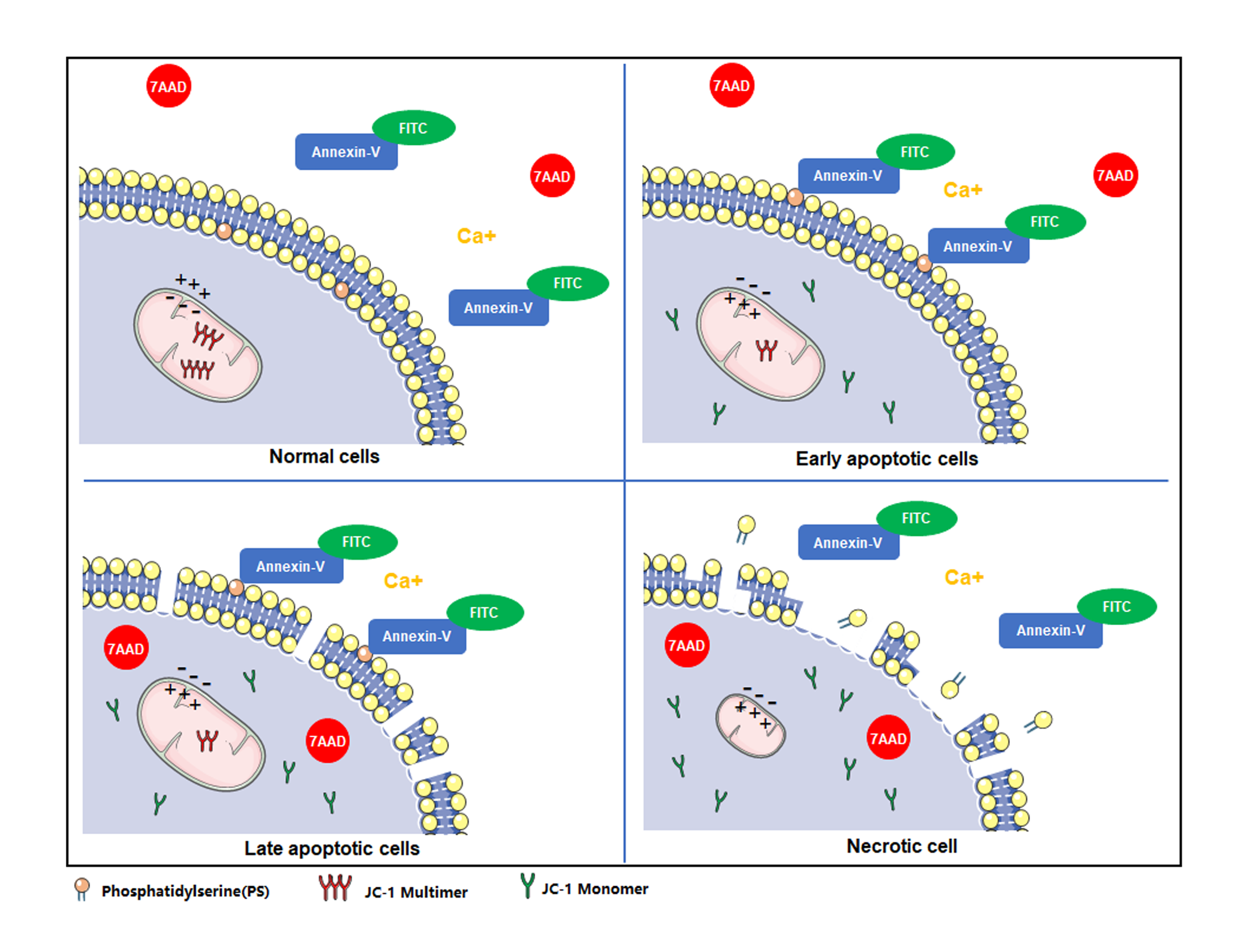

Supplement: Supplementary file 4 — Figure S4 Schematic diagram of cell apoptosis and detection principle of dye combination. Under normal circumstances, the phospholipid is specifically distributed in the inner lobes of the plasma membrane phospholipid bimolecular. The membrane potential of normal cells is stable. JC‐1 enters the mitochondria through the polarity of the mitochondrial membrane and forms a red fluorescent probe due to the increased concentration. Multimer, which is double‐positive for FL1 and FL2.In the early stage of apoptosis, PS flips from the inner lobes of the plasma membrane to the outer lobes of the plasma membrane. Annexin V is a Ca+ dependent phospholipid‐binding protein that can specifically bind to the PS that is flipped to the outer leaf of the plasma membrane with high affinity. FITC‐labelled Annexin V is used as a probe. At the same time, the mitochondrial transmembrane potential is depolarized, and JC‐1 is released from the mitochondria; the concentration is reduced, and it is reversed to a monomer form that emits green fluorescence, which is FL1 single positive. In the middle and late stages of apoptosis and dead cells, 7AAD can pass through the cell membrane and combine with the nucleus to appear red. [file CPR-55-e13300-s005.tif]

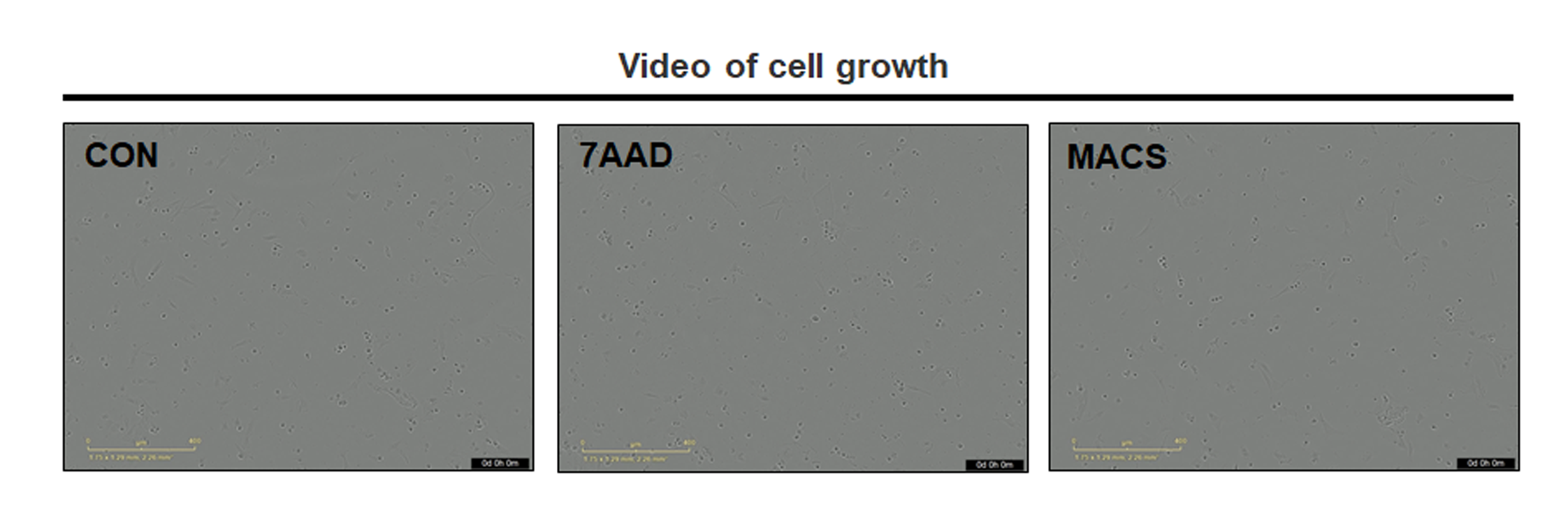

Supplement: Supplementary file 5 — Figure S5 After the three groups of cells were sorted, the growth changes of the cells from 0 to 84 h (Detailed in the supplement Video). After flow sorting, CD106+ hUC‐MSCs from the three treatment groups were seeded and observed in real‐time using a living cell analyser and then took photos per 12 h. This video shows the photos of cells in three groups taken at different time points from 0 to 84 h (scale bar = 400 μm). The 7AAD and MACS groups gradually became denser compared with the CON group. [file CPR-55-e13300-s002.zip › Figure S5.tif]

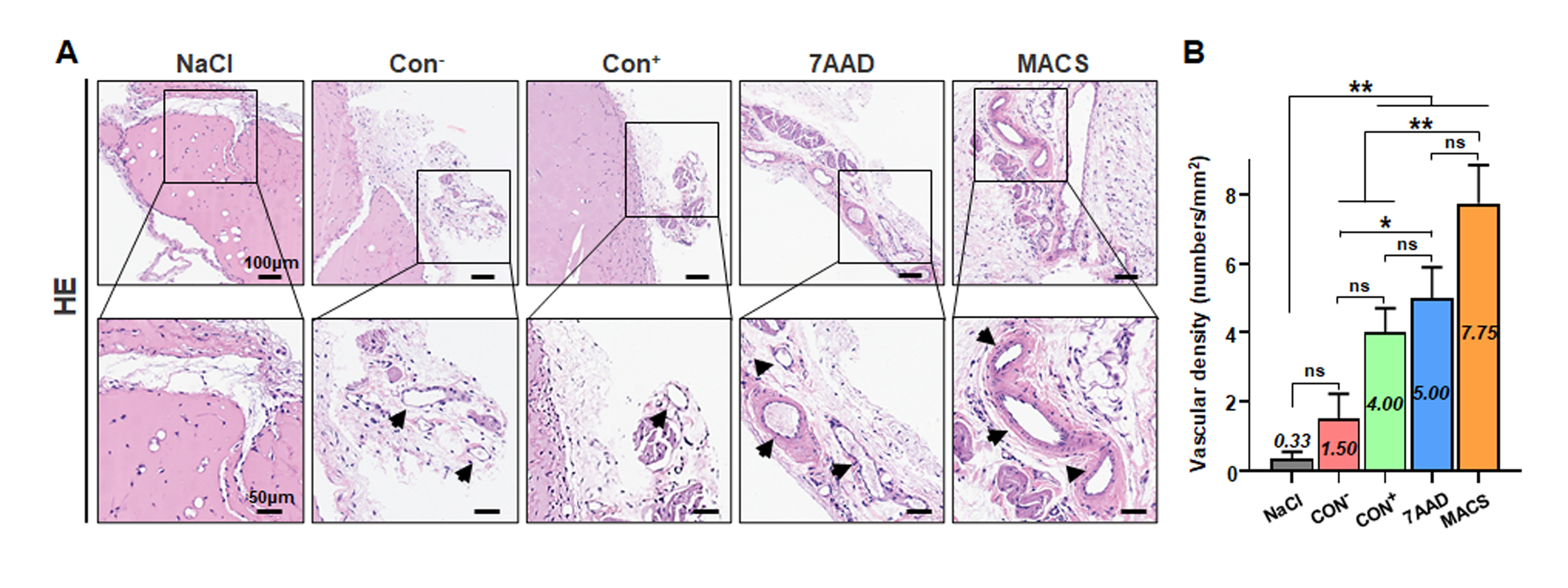

Supplement: Supplementary file 6 — Figure S6 Direct assessment for the vivo vascular‐angiogenic ability of the CD106+ hUC‐MSCs treated by the three methods after flow sorting. (A) Macroscopic and microscopic view of matrigel plugs. The matrigel plug was harvested 21 days later. H & E staining was performed to reveal the vessel density in matrigel plug (scale bar = 100 μm or 50 μm). (B) Quantification of the vascular density of matrigel plugs was performed using Image J software (n = 3/group; all data shown as mean ± SEM). [file CPR-55-e13300-s006.tif]

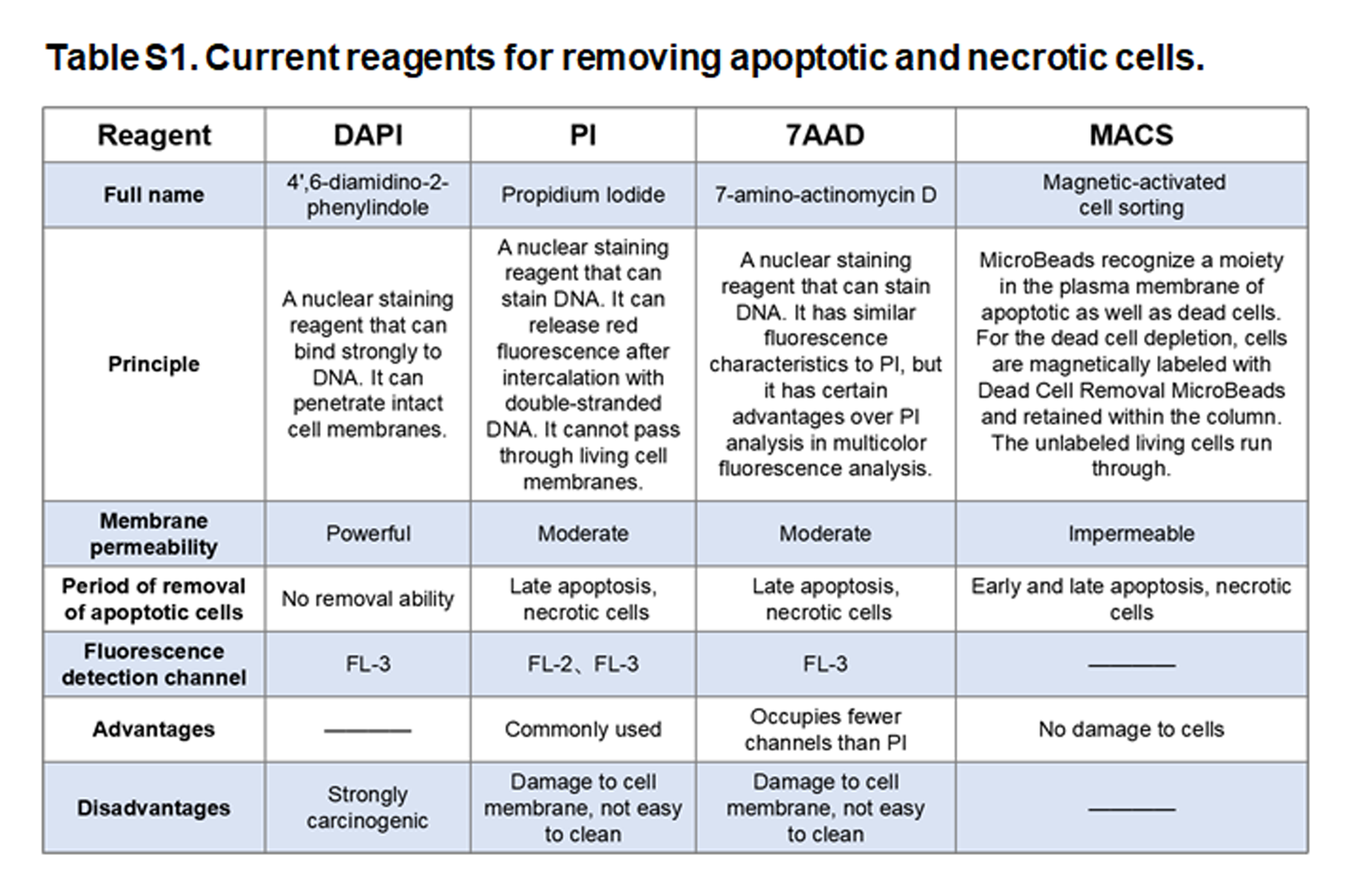

Supplement: Supplementary file 7 — Table S1 Current reagents for removing apoptotic and necrotic cells. [file CPR-55-e13300-s004.tif]
